# Engineering editopes through programmable RNA editing toward tumor neoantigen generation

**DOI:** 10.1038/s44318-026-00870-5

**Published:** 2026-08-14

**Authors:** Riccardo Pecori, Beatrice Casati, Rona Merdler-Rabinowicz, Netanel Landesman, Khwab Sanghvi, Stefan Zens, Ece Bilgin, Mantiana Topouzi, Kai Kipfstuhl, Veronica Pinamonti, Annette Arnold, Mohammad Abukhalaf, David Gomez-Zepeda, Stefan Tenzer, John M Lindner, Michael Platten, Rienk Offringa, Rafael Carretero, Eytan Ruppin, Erez Y Levanon, Fotini Nina Papavasiliou

**Affiliations:** 1 Division of Immune Diversity, German Cancer Research Center (DKFZ), Heidelberg, 69120 Germany; 2 Cancer Data Science Lab, Center for Cancer Research, National Cancer Institute, National Institutes of Health, Bethesda, MD 20892 USA; 3 Mina and Everard Goodman Faculty of Life Sciences, Bar-Ilan University, Ramat Gan, 5290002 Israel; 4 Division of Neuroimmunology and Brain Tumor Immunology, German Cancer Research Center (DKFZ), Heidelberg, 69120 Germany; 5 Department of Neurology, Medical Faculty Mannheim, MCTN, University of Heidelberg, Mannheim, Germany; 6 Division of Molecular Oncology of Gastrointestinal Tumors, German Cancer Research Center (DKFZ), Heidelberg, 69120 Germany; 7 DKFZ Drug Discovery Lab (D3Lab), German Cancer Research Center (DKFZ), Heidelberg, 69120 Germany; 8 Faculty of Biosciences, Heidelberg University, 69120 Heidelberg, Germany; 9 BioMed X Institute, 69120 Heidelberg, Germany; 10 Helmholtz Institute for Translational Oncology (HI-TRON), Mainz, German Cancer Research Center, Mainz, Germany; 11 Division of Immunoproteomics, German Cancer Research Center (DKFZ), Heidelberg, 69120 Germany; 12 Immunopeptidomics Platform, German Cancer Research Center (DKFZ), Heidelberg, 69120 Germany; 13 University Medical Center of the Johannes-Gutenberg University, Mainz, Germany; 14 DKTK (German Cancer Consortium) Clinical Cooperation Unit (CCU) Neuroimmunology and Brain Tumor Immunology, German Cancer Research Center (DKFZ), Heidelberg, Germany; 15 German Cancer Consortium (DKTK), DKFZ, Core Center Heidelberg, Heidelberg, Germany; 16 DKFZ-Hector Cancer Institute at University Medical Center Mannheim, Mannheim, Germany; 17 Department of Biochemistry, molecular Biology 3 and immunology. University of Granada, Granada, Spain

**Keywords:** Cancer, Immunology, RNA Biology

## Abstract

Neoepitope-based therapies hold great promise for cancer immunotherapy because they target tumor-specific mutations and elicit potent anti-tumor T-cell responses. However, their clinical implementation remains limited by the complexity of neoepitope discovery and uncertainty regarding presentation by tumor cells. A potential alternative is the generation of immunogenic neoepitopes directly within cancer cells through programmable RNA editing. Here, we develop Short Precise-Encodable ADAR Recruiting (SPEAR) gRNAs that harness endogenous ADAR1 to direct precise adenosine-to-inosine (A-to-I) editing at selected transcript sites. Using these gRNAs, we demonstrate the generation of immunogenic neoepitopes through RNA editing at the transcript level, termed *editopes*. In a proof-of-concept model based on the melanoma antigen MART-1, SPEAR-mediated RNA editing restored antigen-specific T cell recognition and enabled tumor control in vivo. Finally, we developed a computational pipeline to identify candidate tumor-selective neoepitopes across multiple cancer types amenable to guided RNA editing. Our findings establish programmable RNA editing as a strategy for engineering immunogenic editopes and provide a framework for neoepitope-directed cancer immunotherapy.

## Synopsis

Clinical implementation of neoepitope-based cancer immunotherapy remains limited by challenges with neoepitope identification and presentation by tumor cells. This study develops Short Precise-Encodable ADAR Recruiting (SPEAR) gRNAs, a programmable RNA editing platform that harnesses endogenous ADAR1 to generate immunogenic neoepitopes at the transcript level (“editopes”), and uses them to enable antigen-specific T cell recognition in vitro and tumor control in vivo.

- SPEAR gRNAs enable efficient and precise recruitment of endogenous ADAR1 for programmable RNA editing.
- RNA editing can generate known immunogenic neoepitopes directly at the transcript level, creating “editopes”.
- Editopes restore antigen-specific T cell recognition and activation in vitro.
- SPEAR-mediated editing enables tumor control in vivo following adoptive T cell therapy.
- A computational pipeline identifies candidate tumor-selective neoepitopes amenable to RNA editing across multiple cancer types.

## Abstract (web summary)

The new RNA editing platform enables recruitment of endogenous ADAR1 to generate immunogenic neoepitopes at the transcript level (“editopes”).

## Graphical abstract

**Figure:**
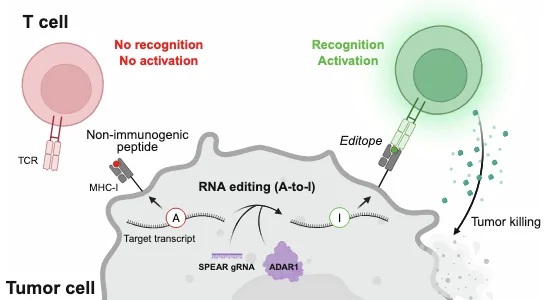

**Figure:**
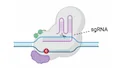

## Introduction

In recent years, immunotherapy has emerged as a revolutionary approach in cancer therapy, demonstrating remarkable clinical response in numerous cases with acceptable patient risk (Esfahani et al, 2020). This success depends on the ability to trigger a robust immune response against the tumor and requires both the presence of cancer-specific antigens on tumor cells and T lymphocytes bearing antigen-specific T cell receptors (TCR) that recognize and kill them. Responsiveness to immunotherapy is positively correlated with tumor mutational burden (TMB) (Chan et al, 2019; Yarchoan et al, 2017; Samstein et al, 2019). Since only a subset of somatic mutations give rise to neoepitopes that are processed and presented as non-self by the major histocompatibility complex (MHC), tumors with a high TMB are more likely to produce immunogenic neoantigens and to respond favorably to immunotherapy (Schumacher and Schreiber, 2015).

Neoantigens arise from non-synonymous DNA mutations, but also from aberrant post-transcriptional and post-translational modifications, including aberrant mRNA translation, alternative splicing, and RNA editing (Nagel et al, 2022; Zhang et al, 2018; Smart et al, 2018). However, many cancers harbor an insufficient repertoire of neoantigens capable of eliciting a strong immune reaction (Bonaventura et al, 2019) and are therefore poorly responsive to immunotherapy. To address this challenge, strategies aimed at artificially enhancing neoantigen production have emerged, such as modulation of alternative splicing (Lu et al, 2021). Yet, these approaches are not tumor-specific and can often also affect healthy tissues, potentially leading to adverse side effects, such as autoimmunity. Effective methods to generate specific neoantigens exclusively within tumor cells are still lacking.

Adenosine deaminase acting on RNA 1 (ADAR1) is an enzyme that recognizes long double-stranded RNA (dsRNA) structures and converts adenosines (A) to inosines (I), which are interpreted as guanosines (G) by the cellular translation machineries (Licht et al, 2019; Porath et al, 2017; Basilio et al, 1962). This attribute has enabled the development of guided RNA editing by recruiting the endogenous enzyme with an antisense guide RNA, a so-called ADAR-engager or ADAR-recruiting gRNA (reviewed in REFs (Casati et al, 2021; Aquino-Jarquin, 2020; Vogel and Stafforst, 2018)). ADAR1 activity, which is already abundant across the mammalian transcriptome (Tan et al, 2017), increases in almost all cancers (Paz-Yaacov et al, 2015), contributing to transcriptomic diversity and neoantigen generation (Zhang et al, 2018).

While ADAR-engagers have been explored in the clinic for the correction of disease-causing mutations such as premature stop codons, we hypothesized that this same mechanism could be repurposed to deliberately introduce precise nucleotide substitutions into cancer cell mRNAs to engineer tumor-specific neoantigens. To this end, we set out to develop Short Precise-Encodable ADAR Recruiting (SPEAR) ADAR-engagers; compact, genetically encodable guide RNAs specifically designed to recruit endogenous ADAR1 and drive A-to-I RNA editing in a highly controlled manner. To evaluate the feasibility of this approach, we selected a well-characterized tumor antigen, MART-1 (melanoma-associated antigen recognized by T cells-1), and we engineered it to generate a non-immunogenic variant as a model system to assess whether targeted A-to-I editing could restore its immunogenicity and, consequently, T cell recognition and cytotoxicity. By applying this proof-of-concept strategy, we aimed to evaluate the potential of RNA editing to induce functional neoantigen formation directly from endogenous transcripts.

Building on this conceptual framework, we also designed a computational pipeline to systematically identify tumor-specific somatic mutations that could serve as substrates for SPEAR-mediated editing. Because these editing events are designed to target only transcripts harboring tumor-specific mutations, the strategy offers the potential for tumor-selective neoantigen formation with minimal risk to healthy tissues. This approach provides a personalized and safe avenue for enhancing tumor immunogenicity and expanding the repertoire of actionable neoantigens for cancer immunotherapy.

## Results

### Optimization of short precise-encodable ADAR1-recruiting (SPEAR) gRNAs

Several methods have enabled precise site-directed RNA editing using engineered or endogenous editing enzymes. A particularly useful approach relies on ADAR-engagers that base-pair with target transcripts to form dsRNA structures capable of recruiting endogenous ADAR1 to specific RNA sites (Booth et al, 2023). This method offers several therapeutic advantages: it eliminates the need for exogenous protein (these programmed oligonucleotides can be delivered directly into cells), reduces the molecular payload (ADAR-engagers consisting of merely a few dozen nucleotides (nt) prove effective variants (Monian et al, 2022; Reautschnig et al, 2022; Qu et al, 2019; Merkle et al, 2019)), and minimizes the risk of an anti-drug immune response (allowing for repeat dosing).

ADAR-engagers can be delivered as chemically modified antisense oligonucleotides (ASOs), as genetically encoded constructs via plasmids or viral particles, or as in vitro transcribed RNA moieties. Upon hybridization with target mRNAs, gRNAs form duplexes recruiting endogenous ADAR enzymes to catalyze A-to-I editing (Reautschnig et al, 2022; Qu et al, 2019; Merkle et al, 2019; Katrekar et al, 2022; Reautschnig et al, 2025; Yi et al, 2022; Byrne et al, 2025; Smargon et al, 2025; Sun et al, 2025). These gRNAs generally fall into two main categories: (1) long gRNAs (>100 nt) with extended antisense “specificity domains” for target pairing (Qu et al, 2019; Katrekar et al, 2022; Yi et al, 2022), and (2) shorter, structured gRNAs that include a compact specificity domain along with a “recruitment domain” RNA motif to enhance ADAR recruitment (Reautschnig et al, 2022; Merkle et al, 2019; Reautschnig et al, 2025; Katrekar et al, 2019). Long gRNAs are prone to extensive bystander off-target editing, requiring time-consuming, target-specific optimization (Qu et al, 2019; Katrekar et al, 2022; Yi et al, 2022). In contrast, short, structured gRNAs exhibit high precision and editing efficiency when chemically modified and delivered as ASOs (Monian et al, 2022; Merkle et al, 2019), but show poor performance when genetically encoded (Wettengel et al, 2017). More complex designs using clustered ADAR recruitment motifs offer improved specificity, though they involve intricate design rules and require non-intuitive computational tools (Reautschnig et al, 2022, 2025). To overcome these challenges, we sought to optimize a genetically encodable gRNA that could efficiently and precisely recruit endogenous ADAR1. Our goal was to minimize the gRNA structure required for efficient ADAR1 recruitment, thereby developing a simple and flexible platform for therapeutic deployment. Such optimized gRNAs could be delivered as plasmid- or virus-encoded constructs, or alternatively as chemically stabilized RNA molecules for direct administration.

**Figure 1:**
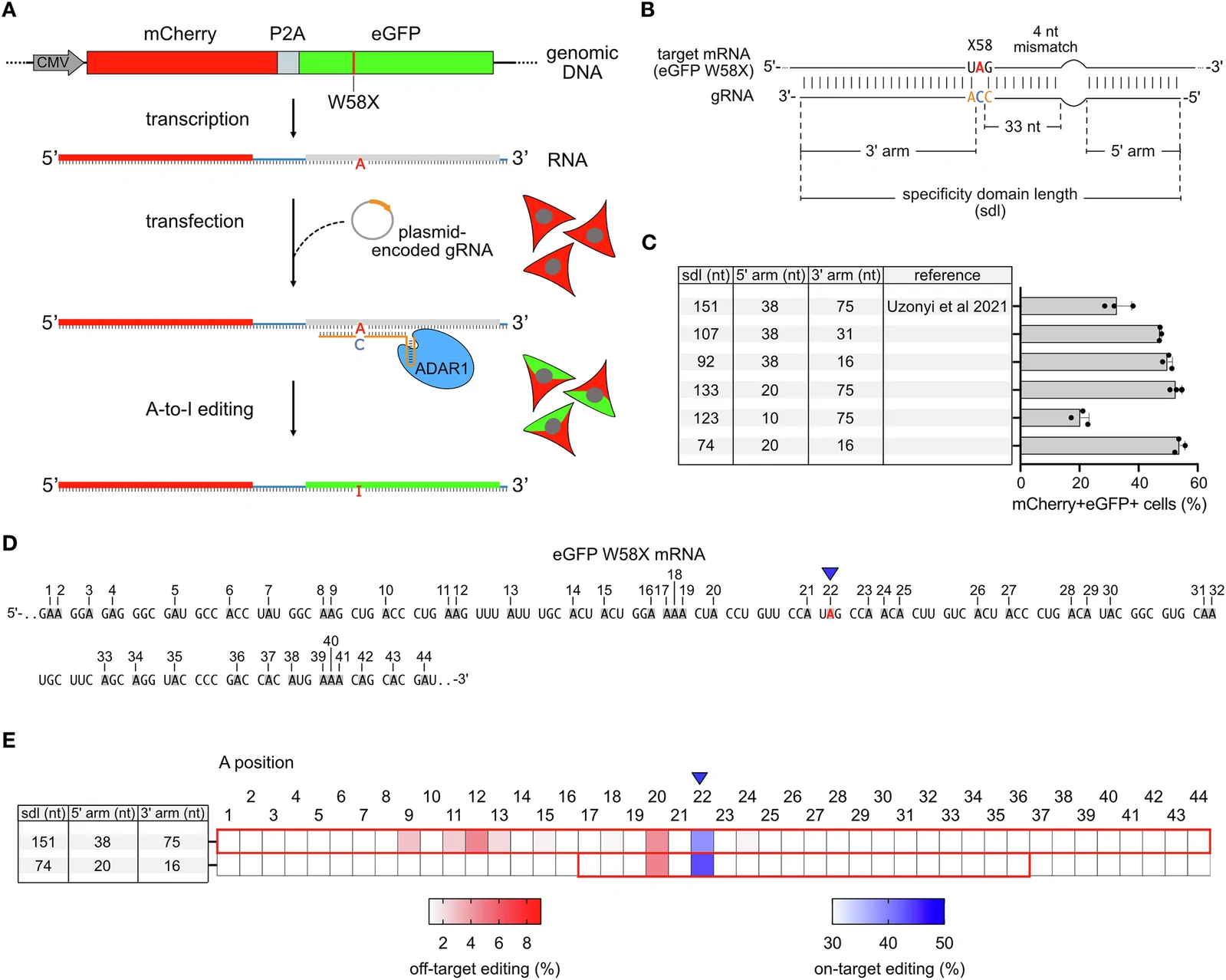
Short precise-encodable ADAR recruiting (SPEAR) gRNAs enable efficient and precise site-directed RNA editing. (**A**) Schematic of the dual-reporter cell line and the experimental setup used to screen different genetically encoded gRNAs. (**B**) Diagram of the eGFP^W58X^ target mRNA and the corresponding targeting gRNA used in the experiment shown in panel (**C**). (**C**) Flow cytometry analysis of mCherry and eGFP double-positive cells, presented as a bar plot for the tested gRNAs. (**D**) Schematic of the sequencing window where A-to-I edits were assessed, as shown in panel (**E**). (**E**) Heatmap of A-to-I editing from NGS amplicon sequencing, showing on-target editing (in blue) and off-target editing (in red). The region targeted by each gRNA is indicated with a red rectangle. For panel C, bars represent the mean for three biological replicates; error bars indicate the standard deviation. Source data are available online for this figure.

To evaluate the capability of such gRNAs to recruit endogenous ADARs, we generated a dual-reporter HEK293T cell line stably expressing mCherry and a mutated eGFP containing a premature stop codon (W58X) linked by a P2A self-cleaving peptide (Liu et al, 2017). In the absence of editing, only mCherry is expressed. Successful recruitment of ADAR to the UAG codon converts this stop codon to UIG, which is interpreted as a tryptophan codon (UGG), thereby restoring eGFP fluorescence (Fig. 1A and *REF* (Montiel-González et al, 2016)). This system enabled rapid, quantitative assessment of gRNA activity via flow cytometry. Additionally, HEK293T cells predominantly express ADAR1 with minimal ADAR2 expression (Qu et al, 2019), allowing us to specifically assess ADAR1-dependent editing. Notably, the use of a stably integrated reporter mimics the behavior of an endogenous transcript more accurately than co-transfection of guide and target RNAs, thereby offering a more realistic and relevant testing environment.

**Figure EV1:**
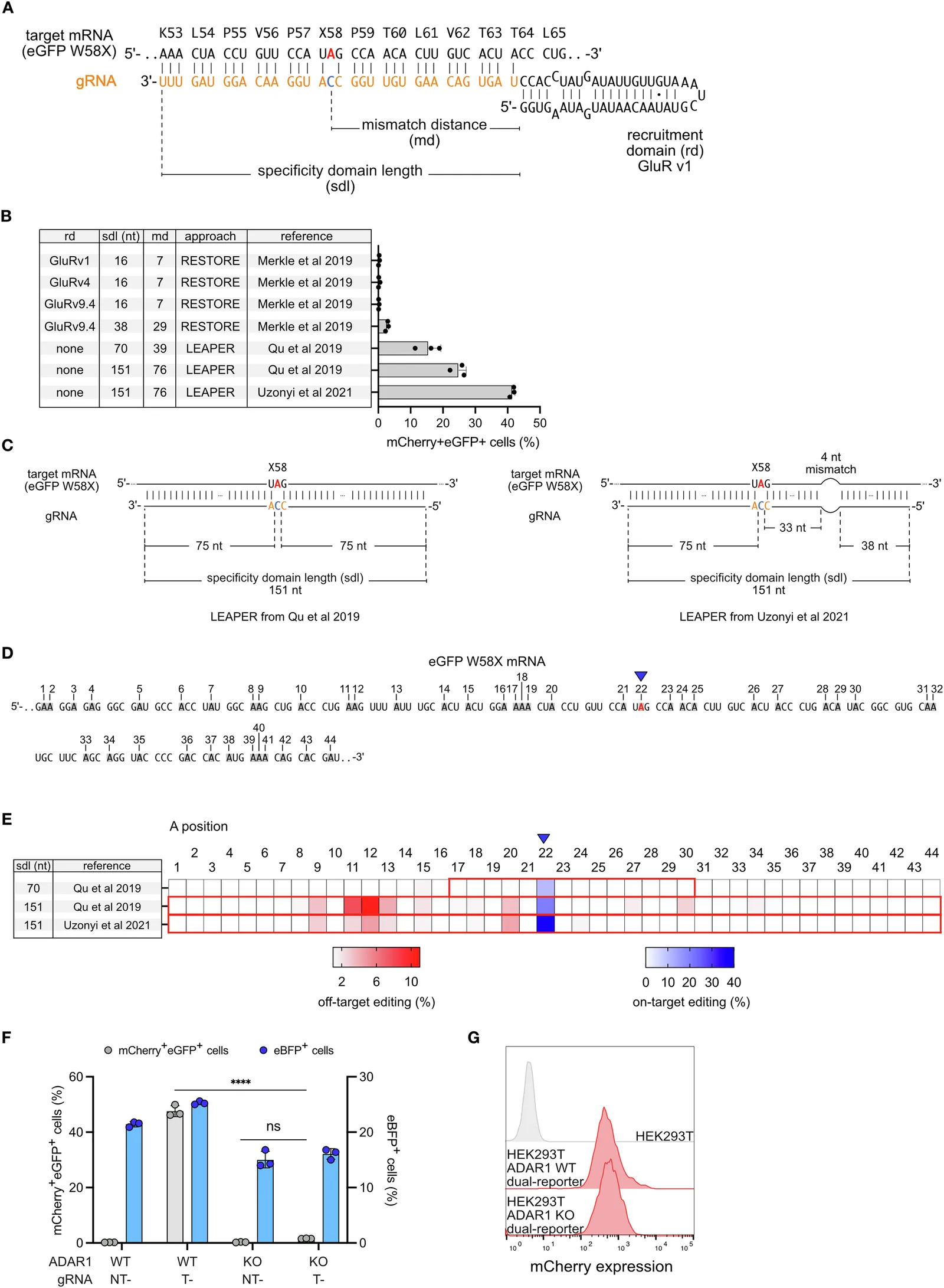
Optimization of Short Precise-Encodable ADAR Recruiting (SPEAR) gRNAs. (**A**) Schematic of the eGFP^W58X^ target mRNA and the corresponding targeting gRNAs used in the experiment shown in panel (**B**). (**B**) Flow cytometry analysis of mCherry and eGFP double-positive cells, presented as a bar plot for the tested gRNAs. Bars represent the mean for three biological replicates; error bars indicate the standard deviation. (**C**) Diagram illustrating the difference between LEAPER gRNAs without (left (Qu et al, 2019)) or with a 4-nucleotide (nt) mismatch (right (Uzonyi et al, 2021)). (**D**) Schematic of the sequencing window where A-to-I edits were assessed, as shown in panel (**E**). (**E**) Heatmap of A-to-I editing from NGS amplicon sequencing, showing on-target editing (in blue) and off-target editing (in red) for three different LEAPER gRNAs. The region targeted by each gRNA is indicated with a red rectangle. (**F**) Flow cytometry analysis of mCherry- and eGFP double-positive cells (left, gray bars) and eBFP-positive cells (right, blue bars), presented as a bar plot for the optimized SPEAR gRNA in HEK293T ADAR1 WT and KO dual-reporter cell lines. Bars represent the mean for three biological replicates; error bars indicate the standard deviation (adjusted *p* values: **** <0.0001, ns = 0.3928, two-way ANOVA with multiple comparisons). (**G**) Flow cytometry analysis of mCherry expression of HEK293T ADAR1 WT and KO dual-reporter cell lines, used in the experiment shown in panel (**F**).

We began by examining the impact of three structural features of encoded gRNAs: (1) specificity domain length, (2) the distance between the target adenosine and the recruitment domain, and (3) the recruitment domain itself (Fig. EV1A). Consistent with prior work, we confirmed that long, unstructured gRNAs were more effective than structured gRNAs when expressed from plasmids (Fig. EV1B), and that editing efficiency improved upon inclusion of a 4-nt mismatch placed 35 nt downstream of the target adenosine (Uzonyi et al, 2021) (Fig. EV1B,C). However, amplicon sequencing revealed that longer gRNAs incurred greater off-target editing across adjacent adenosines, validating concerns about precision and bystander activity (Fig. EV1D,E). This analysis also confirmed the flow cytometry results for on-target editing, validating the robustness of our dual-reporter cell line for gRNA screening (Fig. EV1E).

To systematically optimize gRNA architecture, we designed a series of truncated gRNAs based on the long LEAPER construct with a 4-nt mismatch. We varied the length of the 5′ and 3′ arms independently (Fig. 1B,C). Reducing the 3′ arm from 75 to 16 nt resulted in a 1.5-fold increase in mCherry^+^eGFP^+^ cells. Truncating the 5′ arm (externally of the 4-nt mismatch) from 38 to 20 nt resulted in a modest gain in editing, while further shortening to 10 nt drastically reduced activity (Fig. 1C). These findings suggest that the duplex formed by the 5’ arm of the gRNA is essential for ADAR1 recruitment and positioning. Combining both optimizations, we generated a compact 74-nt-long gRNA with reduced 5′ and 3′ arms, half the size of the original 151-nt LEAPER. This shortened design yielded the highest editing efficiency, as measured by fluorescence restoration (Fig. 1C), and exhibited reduced off-target editing, as determined by amplicon sequencing (Fig. 1D,E).

To directly confirm the identity of the editing enzyme in this system, we repeated the dual-reporter assay in a HEK293T ADAR1 knockout cell line we previously generated (Pecori et al, 2023). Cells were transfected with the optimized 74-nt-long gRNA expressed from a backbone encoding eBFP, allowing quantification of transfection efficiency via eBFP expression. Editing activity, measured as restoration of eGFP fluorescence, was observed exclusively in ADAR1 wild-type cells and was completely abolished in ADAR1 knockout cells, despite effective plasmid transfection in both cell lines (WT: 25.3% eBFP⁺ cells, SD = 0.3%; KO: 16.1% eBFP⁺ cells, SD = 0.9%; Fig. EV1F). To exclude potential bias due to differences in reporter expression, we used matched dual-reporter cell lines with comparable mCherry expression levels in ADAR1 wild-type and knockout backgrounds (Fig. EV1G). These results demonstrate that RNA editing in this system is strictly ADAR1-dependent and confirm that the optimization shown here is specific to ADAR1.

Collectively, these results demonstrate that the efficiency and precision of encodable ADAR-engagers can be significantly improved through rational design of duplex geometry. Specifically, we identify the length of the duplex region downstream of the 4-nt mismatch as a key determinant of ADAR1 recruitment. We refer to this optimized design as Short Precise-Encodable ADAR Recruiting (SPEAR) gRNAs.

### SPEAR gRNAs enable the generation of immunogenic neoantigens at the RNA level (editopes)

**Figure 2:**
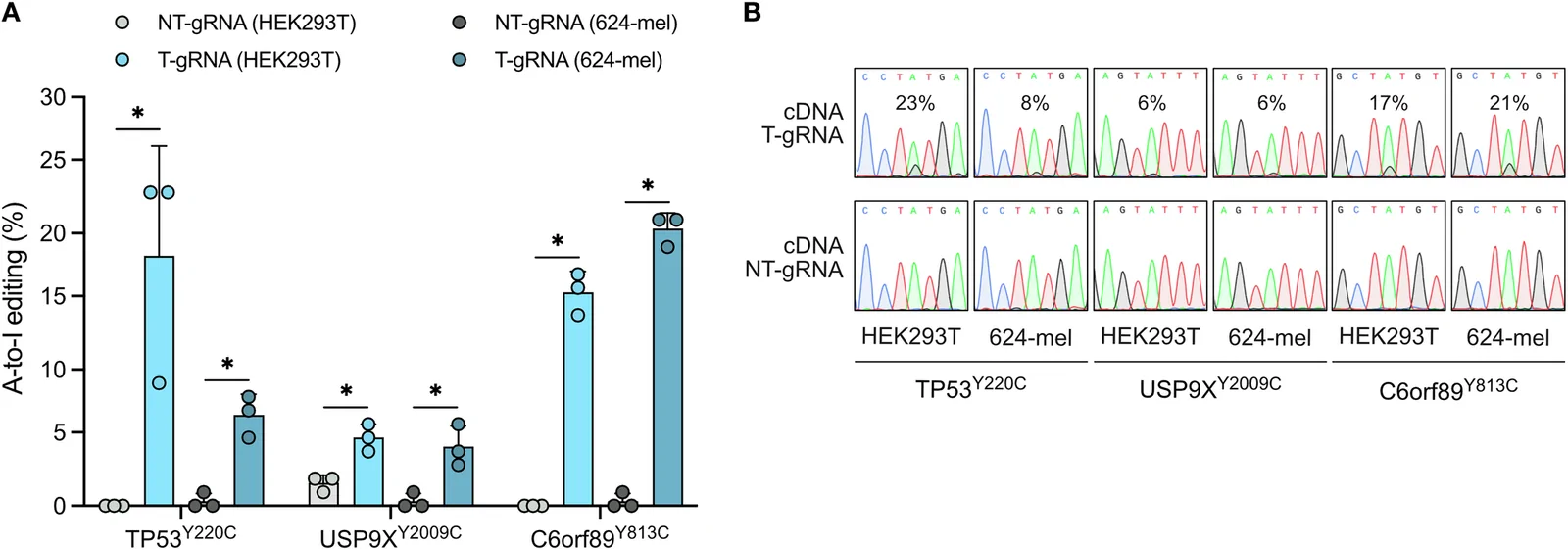
SPEAR gRNAs enable the generation of known immunogenic neoepitopes at the RNA level. (**A**) Bar plot showing A-to-I editing percentages at selected endogenous target sites corresponding to known immunogenic neoepitopes (TP53^Y220C^, USP9X^Y2009C^, and C6orf89^Y813C^) in HEK293T and 624-mel cells expressing either targeting (T) or non-targeting (NT) SPEAR gRNAs. Editing was quantified by Sanger sequencing using MultiEditR (Kluesner et al, 2021). Bars represent the mean of three biological replicates; error bars indicate the standard deviation. Statistical significance was assessed using multiple unpaired t-tests with Holm–Šídák correction (adjusted *p* values: *TP53 HEK293T = 0.017124, *TP53 624-mel = 0.005107, *USP9X HEK293T = 0.014925, *USP9X 624-mel = 0.013236, *C6orf89 HEK293T = 0.000177, *C6orf89 624-mel = 0.000034). (**B**) Representative Sanger sequencing traces from HEK293T and 624-mel cells expressing T- or NT-gRNAs, showing A-to-I editing at the indicated target sites. Percent editing at each site is indicated above the traces. Source data are available online for this figure.

To determine whether SPEAR gRNAs can generate known immunogenic neoantigens at the RNA level, we selected a set of endogenous target sites corresponding to previously described cancer-associated neoepitopes. Specifically, TP53^Y220C^, USP9X^Y2009C^, and C6orf89^Y813C^ mutations have been reported to give rise to immunogenic neoantigens in ovarian, head and neck cancers (Deniger et al, 2018; Ito et al, 2007) and chronic lymphocytic leukemia (Rajasagi et al, 2014), respectively. These sites were chosen to test whether RNA editing could recapitulate, at the transcript level, amino acid substitutions known to generate T cell-recognizable peptides. For each target, we designed SPEAR gRNAs to direct A-to-I editing at specific adenosines in the corresponding transcripts, thereby introducing defined coding changes that are expected to generate the desired neoepitopes. We evaluated editing efficiency in both HEK293T and 624-mel cells transfected with plasmids encoding either targeting (T) or non-targeting (NT) SPEAR gRNAs. Sanger sequencing analysis of cDNA revealed reproducible A-to-I editing at all tested sites in cells expressing the corresponding T-gRNAs, while no editing was detected in NT controls (Fig. 2A,B).

These results indicate that SPEAR-mediated RNA editing is not limited to restoring disrupted epitopes in a model antigen system (MART-1) but can also be used to generate known immunogenic neoepitopes directly at the RNA level. Together, these findings establish that SPEAR gRNAs provide a generalizable approach for the programmable generation of immunogenic neoantigens at the RNA level, which we named editopes. These findings prompted us to investigate whether SPEAR-generated editopes could functionally restore antigen recognition and mediate T cell-dependent tumor targeting.

### SPEAR-generated editopes restore antigen-specific T cell activation

Having established that SPEAR gRNAs can generate known immunogenic neoepitopes at the RNA level, we next asked whether such editopes could functionally restore antigen-specific T cell activation. To test this aspect, we designed a proof-of-concept experiment based on the well-characterized melanoma antigen MART-1 (Melanoma Antigen Recognized by T cells-1) (Kawakami et al, 1994; Coulie et al, 1994). MART-1 is encoded by the *MLANA* gene and is a clinically relevant immunogenic target in melanoma. Critically for our purposes, the specific TCR recognizing the MART-1 epitope (EAAGIGLTV) is well characterized. For this, we used the DMF5 TCR, known for its high avidity against MART-1-expressing cells (Johnson et al, 2006).

**Figure 3:**
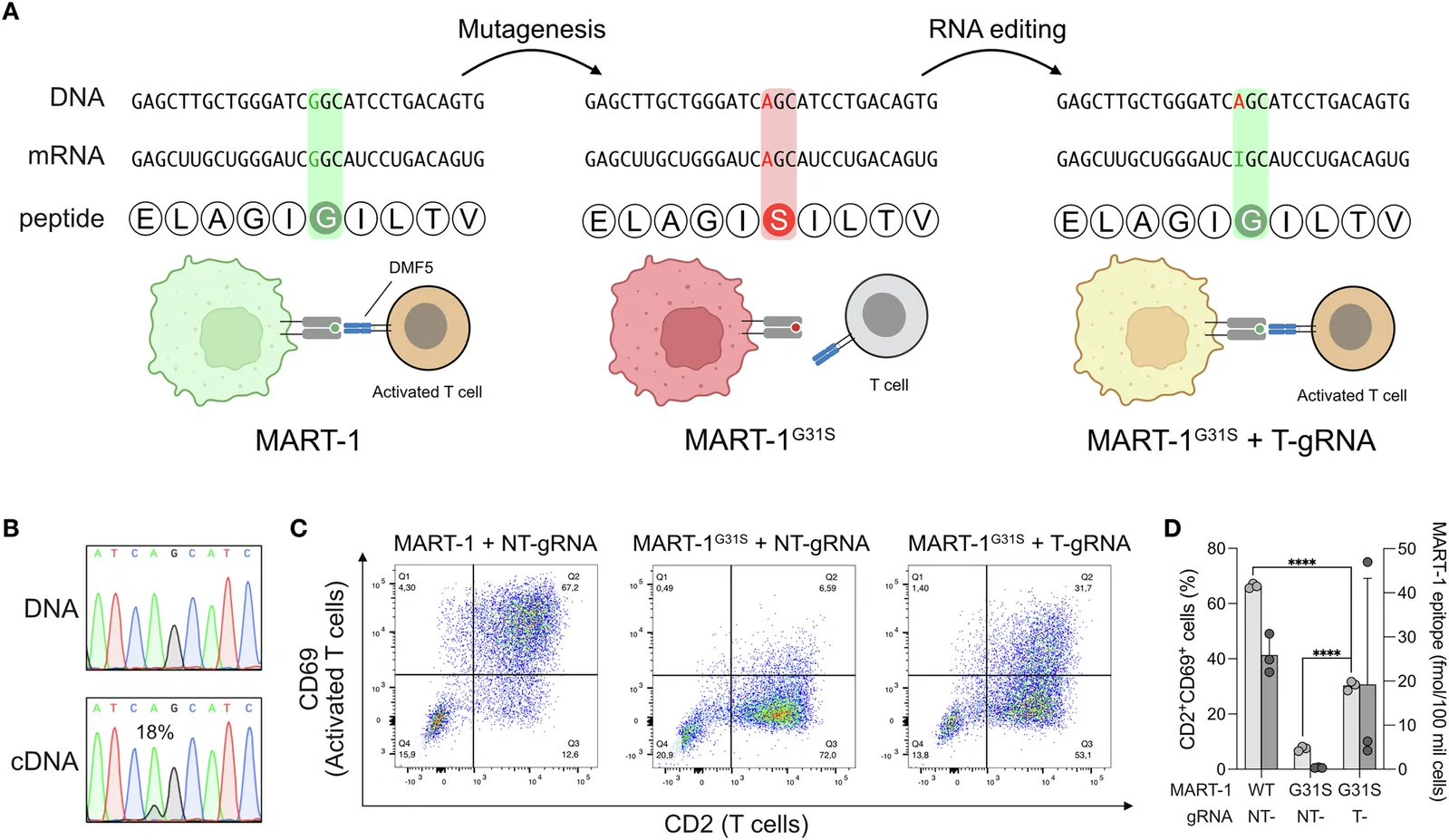
SPEAR gRNAs can generate functional epitopes and activate specific T cells. (**A**) Schematic of the proof-of-principle experiment: HEK293T cells expressing either wild-type (WT) or G31S mutant MART-1 are cocultured with DMF5-expressing T cells, followed by assessment of T cell activation via flow cytometry. SPEAR gRNAs are used to restore the original MART-1 coding sequence at the RNA level, thereby rescuing antigen-specific T cell activation. (**B**) Representative Sanger sequencing traces at the G31S codon from both genomic DNA and cDNA of HEK293T MART-1 G31S cells expressing the SPEAR gRNA. (**C**) Representative flow cytometry dot plots showing CD69 expression as a marker of T cell activation under the three experimental conditions. (**D**) Bar plot showing average T cell activation (left, light gray), measured as the percentage of CD69⁺ CD2⁺ double-positive cells, and the absolute concentration of the presented MART-1^WT^ peptide (right, dark gray) measured with targeted LC–MS immunopeptidomics, across three biological replicates for each condition (adjusted *p* values: **** <0.0001, one-way ANOVA with multiple comparisons). Bars represent the mean for three biological replicates; error bars indicate the standard deviation. Source data are available online for this figure.

**Figure EV2:**
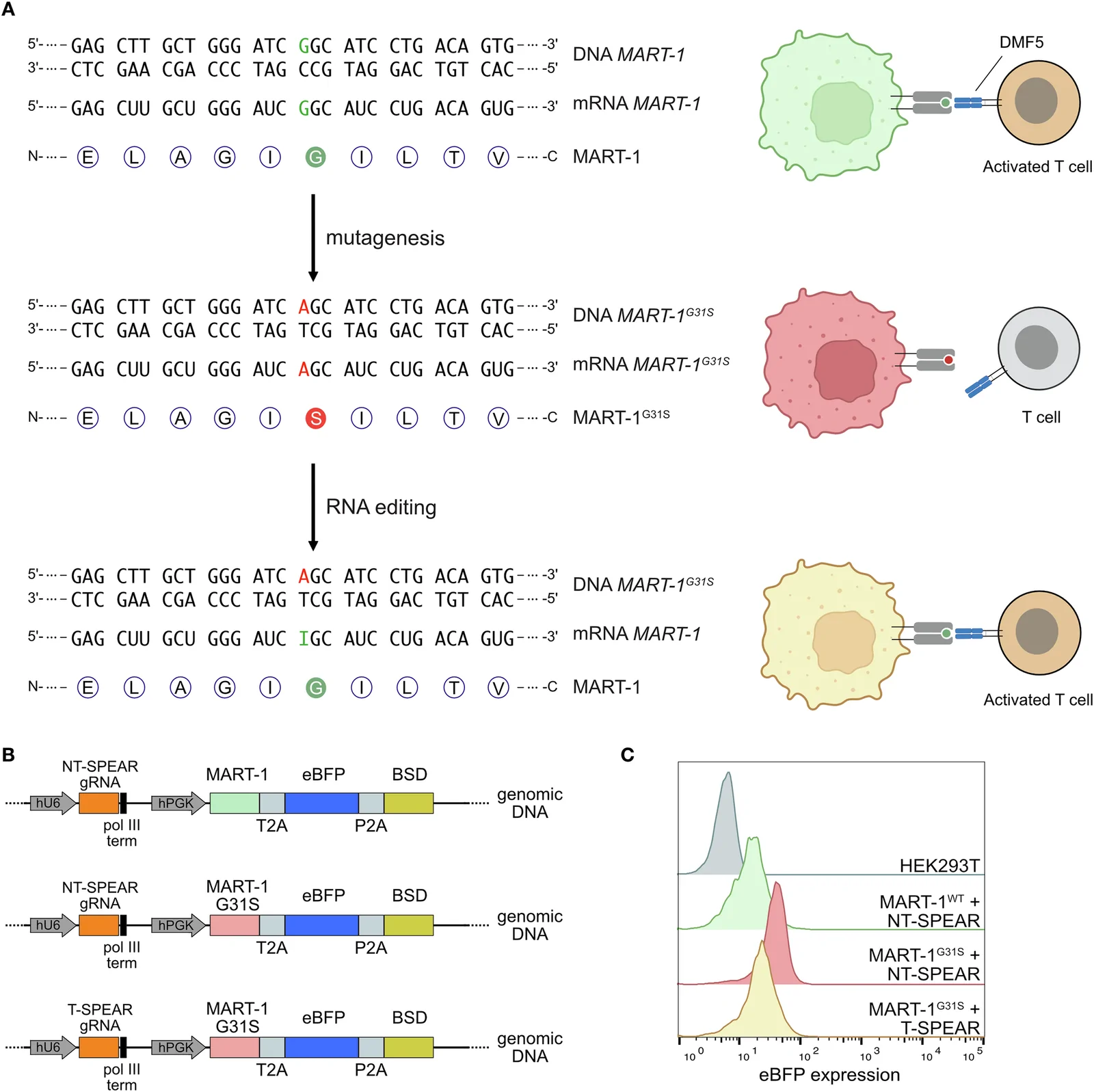
In vitro T cell activation using SPEAR gRNAs. (**A**) Diagram illustrating the molecular setup (left) and the predicted outcome of the cellular coculture (right) corresponding to the experiment presented in Fig. 2. (**B**) Schematic of the constructs used to generate HEK293T cells expressing either wild-type (WT) or G31S mutant MART-1, along with either a non-targeting (NT) or targeting (T) SPEAR gRNA. eBFP was used as a reporter for MART-1 (WT or G31S) expression, and a blasticidin resistance gene (BSD) was used for selecting transfected cells. (**C**) Flow cytometry analysis of eBFP expression for the different cell lines.

To increase T cell sensitivity and stability of the peptide-MHC complex, we used a MART-1 peptide variant (E**L**AGIGILTV) (Valmori et al, 1998) (Figs. 3A and EV2A). We then introduced a point mutation in the MART-1 coding sequence to generate the G31S mutant (MART-1^G31S^; ELAGI**S**ILTV), substituting glycine (**G**GC) with serine (**A**GC) at position 31. This mutation disrupted the epitope, rendering it incapable of activating DMF5 T cells (Figs. 3A,C and EV2A). To reverse this effect via RNA editing, we designed a SPEAR gRNA targeting the mutant *MART-1*^*G31S*^ transcript to recruit endogenous ADAR1 and direct A-to-I editing at the first base of the serine codon (**A**GC), converting it to IGC (interpreted by the ribosome as GGC) at the RNA level and restoring the original epitope (Figs. 3A and EV2A). This system models our broader goal of generating immunogenic editopes from non-immunogenic epitopes through precise RNA editing.

To evaluate whether SPEAR-driven ADAR1 editing could restore antigenicity and T cell activation, we engineered HEK293T and TCR-deficient Jurkat cell lines to stably express either *MART-1*^*WT*^ or *MART-1*^*G31S*^, and the DMF5 TCR, respectively. HEK293T cells were also expressing either a non-targeting (NT) or targeting (T) SPEAR gRNA (Fig. EV2B,C). Sanger sequencing of PCR amplicons from genomic DNA and cDNA in *MART-1*^*G31S*^ cells expressing the T-gRNA confirmed A-to-I editing at the intended site, with an editing efficiency of ~20% (Fig. 3B). We then cocultured the engineered HEK293T and Jurkat cells and assessed T cell activation via flow cytometry using CD2 as a T cell marker and CD69 as an activation marker. As expected, cells expressing *MART-1*^*WT*^ with an NT-gRNA elicited robust T cell activation (~65% CD2^+^CD69^+^ cells), while *MART-1*^*G31S*^ cells with NT-gRNA showed minimal activation (~5%). Importantly, cocultures with *MART-1*^*G31S*^ cells expressing the T-gRNA led to a significant restoration of T cell activation (~30% CD2^+^CD69^+^ cells) (Fig. 3C,D). This was further confirmed by measuring the abundance of the presented *MART-1*^*WT*^ epitope in these cells using targeted LC–MS Immunopeptidomics (Fig. 3D). While the epitope was detected in cells expressing *MART-1*^*WT*^ but not in *MART-1*^*G31S*^ cells in the presence of the NT-gRNA, presentation was recovered in *MART-1*^*G31S*^ cells expressing the T-gRNA at a median of 25.5% compared to *MART-1*^*WT*^ cells.

Despite the moderate level of editing, recovery of T cell activation was substantial (~50%) relative to the wild-type epitope, suggesting that even modest RNA editing rates (<30%) may be sufficient to elicit strong T cell responses and potentially therapeutic immune activation. These results demonstrate that SPEAR gRNAs can efficiently harness endogenous ADAR1 to generate immunogenic peptides capable of activating a specific T cell response. Our findings establish a mechanistic foundation for using programmable RNA editing to engineer *editope* formation in tumor cells.

### Programmable RNA editing drives T cell-mediated tumor rejection in an in vivo mouse model

**Figure EV3:**
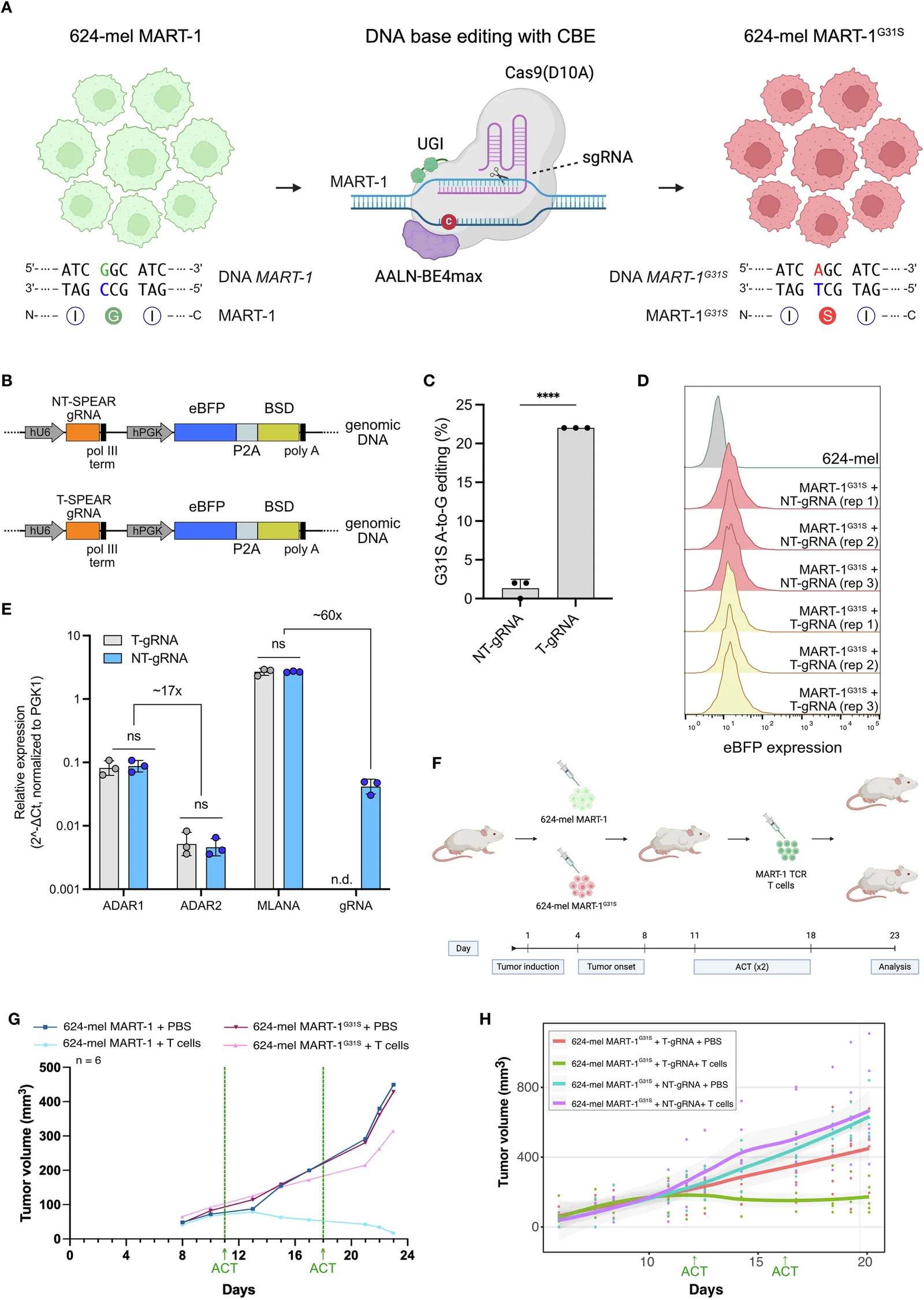
Generation of 624-mel mutant and edited cell lines for evaluating T cell activation and tumor cell elimination in vivo. (**A**) Schematic of the procedure used to generate 624-mel cell clones endogenously expressing MART-1^G31S^. The cytosine base editor AALN-BE4max(Doman et al, 2020) was used to target the cytosine (C highlighted in blue) on the template strand of DNA, opposite the first guanine (G highlighted in green) in the GGC codon encoding glycine (G) at position 31 of the *MART-1* gene. C-to-U base editing converted the codon to AGC, resulting in a glycine-to-serine substitution (G31S). (**B**) Schematic of the constructs used to generate 624-mel MART-1^G31S^ cells expressing either a non-targeting (NT) or targeting (T) SPEAR gRNA. eBFP was used as a reporter for plasmid integration, and a blasticidin resistance gene (BSD) was used for selection. (**C**) Bar plot showing average A-to-I editing percentages at the MART-1 G31S site, quantified from Sanger sequencing traces using MultiEditR (Kluesner et al, 2021) in 624-mel MART-1^G31S^ cells transiently transfected with the plasmids shown in **B** (*p* value: **** <0.0001, two-tailed unpaired *t*-test). Bars represent the mean for three biological replicates; error bars indicate the standard deviation. (**D**) Flow cytometry analysis of eBFP expression in 624-mel MART-1^G31S^ expressing either NT- or T-SPEAR gRNAs, used in the experiments shown in Fig. 4. (**E**) Bar plot representing the expression of ADAR1, ADAR2, MLANA and gRNA measured by RT-qPCR in the 624-mel MART-1^G31S^ lines expressing either NT- or T-SPEAR gRNAs. Relative expression is normalized to PGK1. A two-tailed unpaired *t*-test with Welch’s correction was used to compare the differences in delta Ct values. ns = not significant, center = mean and error bars = standard deviation, *N* = 3. (**F**) Schematic of the in vivo experimental setup for the results shown in (**G**). (**G**) Tumor growth analysis of 624-mel cells expressing MART-1 or MART-1^G31S^ with or without adoptive cell therapy (ACT) using MART-1-specific human-derived T cells. (**H**) Alternative visualization of the in vivo tumor growth shown in Fig. 4E. Each curve represents the mean of six mice, and every single dot represents each mouse.

**Figure 4:**
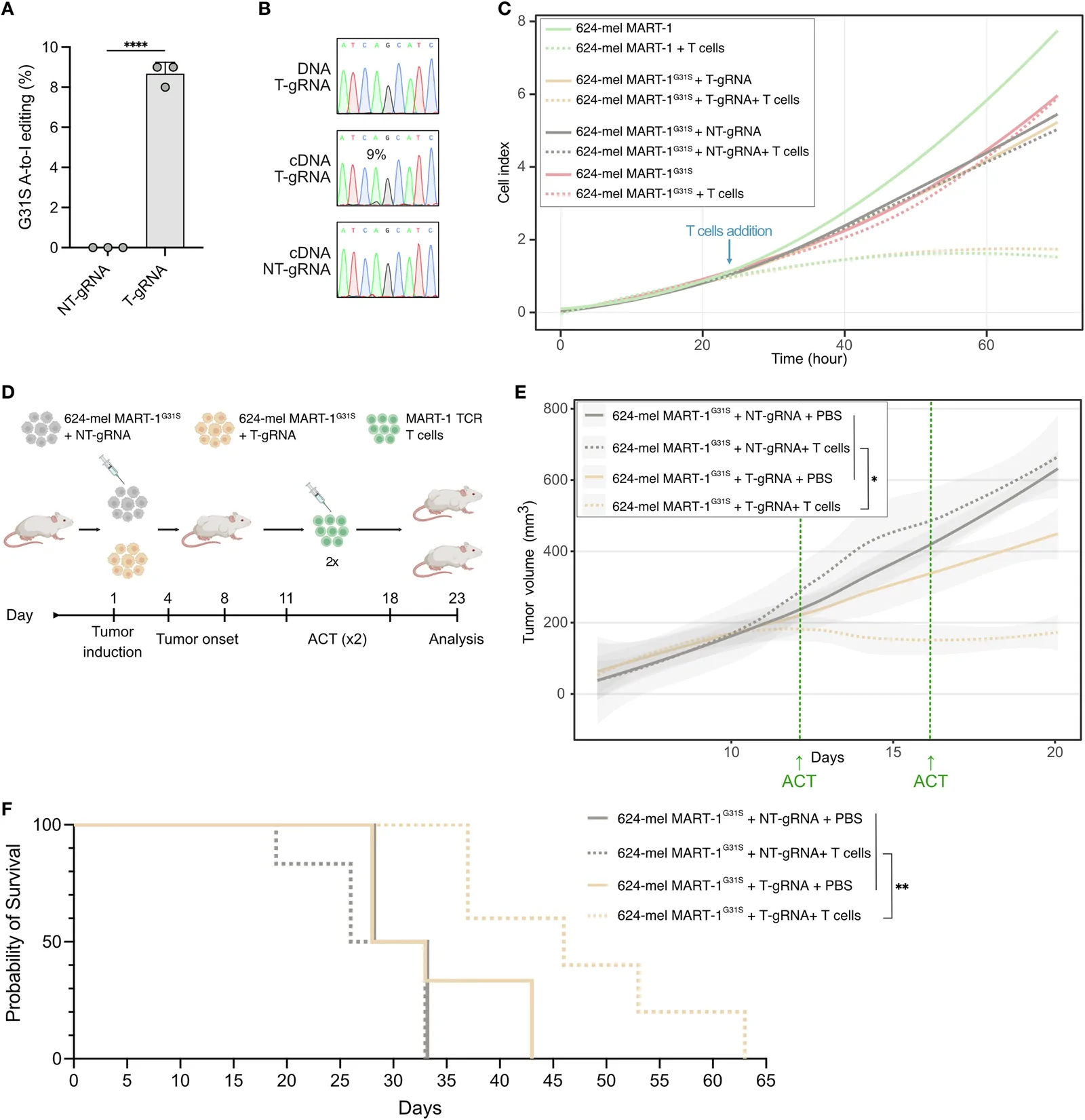
SPEAR gRNA editing induces T cell activation and tumor cell elimination in vivo. (**A**) Bar plot showing average A-to-I editing percentages at the MART-1 G31S site, quantified from Sanger sequencing traces using MultiEditR (Kluesner et al, 2021), in 624-mel MART-1^G31S^ cell lines expressing either a non-targeting (NT) or targeting (T) gRNA (*n* = 3, *p* value: **** <0.0001, two-tailed unpaired *t*-test). Bars represent the mean for three biological replicates; error bars indicate the standard deviation. (**B**) Representative Sanger sequencing traces at the G31S codon from both genomic DNA and cDNA of 624-mel MART-1^G31S^ cells expressing NT- or T-gRNAs. (**C**) xCELLigence real-time analysis of the cytotoxicity of MART-1-specific human T cells cocultured with 624-mel cells expressing MART-1, MART-1^G31S^, or mutant MART-1^G31S^ with NT- or T-gRNAs. For MART-1 or MART-1^G31S^ cells, each curve represents the mean of three technical replicates. For cells expressing NT- or T-gRNAs, each curve represents the mean from three biological replicates, each of those with three technical replicates. T cells were added after 24 h; the cell index is normalized to the time point of T cell addition. (**D**) Schematic of the in vivo experimental setup. (**E**) In vivo tumor growth analysis of MART-1^G31S^ cells expressing the T- or NT-gRNA with or without adoptive cell therapy (ACT) using MART-1-specific human-derived T cells. Each curve represents the mean of six biological replicates. An ordinary one-way ANOVA was applied: * (*P* value: 0.0493). (**F**) Kaplan–Meier survival analysis curves of mice injected with MART-1^G31S^ cells expressing the T- or NT-gRNA with or without adoptive T cell therapy (ACT) using MART-1-specific human-derived T cells. *n* = 6, Logrank (Mantel–Cox) test: ** (*P* value: 0.0055). Source data are available online for this figure.

Having demonstrated that SPEAR-mediated RNA editing can restore cognate T cell activation in vitro, we next evaluated its therapeutic potential in vivo using a xenograft mouse model. For this, we employed the human melanoma cell line 624-mel, which endogenously expresses MART-1. We first introduced the same G-to-A mutation described in the previous section into the endogenous *MLANA* gene using DNA base editing, resulting in expression of the mutant MART-1^G31S^ from the endogenous locus (Fig. EV3A). These cells were further engineered using the Sleeping Beauty system (Cui et al, 2002) to stably express either a targeting (T) or a non-targeting (NT) SPEAR gRNA designed to recruit endogenous ADAR1 to MART-1^G31S^ (Fig. EV3B–D). RT-qPCR analysis confirmed robust expression of the SPEAR gRNA driven by the U6 promoter (Fig. EV3E). While only the NT-gRNA could be reliably detected due to technical limitations, both NT- and T-gRNAs are expressed from the same transposon backbone and showed comparable integration efficiencies, as assessed by eBFP expression (Fig. EV3B,D), supporting similar expression levels. Comparison of gRNA and MLANA transcript levels further revealed that the gRNA is expressed at substantially lower levels (~60-fold) than the target transcript. In addition, stable expression of T- or NT-SPEAR gRNAs did not alter the mRNA expression levels of *MLANA* (*MART-1*), *ADAR1*, or *ADAR2* (Fig. EV3E). Notably, *ADAR1* mRNA expression was ~17-fold higher than *ADAR2* in 624-mel cells (Fig. EV3E), indicating that ADAR1 is likely the predominant editing enzyme in this system, although a contribution from ADAR2 cannot be excluded. We initially assessed editing efficiency in these modified cell lines and observed ~10% A-to-I editing at the endogenous target site in the cell line expressing MART-1^G31S^ together with the T-gRNA, while no editing was detected in cells expressing the NT-gRNA (Fig. 4A,B). To test whether this level of editing was sufficient to induce T cell activation, we performed an in vitro T cell-mediated cytotoxicity assay using the xCELLigence system. As expected, 624-mel MART-1^WT^ cells grew normally in the absence of T cells, but the addition of MART-1-specific T cells resulted in a strong antitumoral effect (Fig. 4C, green lines). In contrast, MART-1^G31S^ cells with no or the control NT-gRNA were unaffected by T cell addition, indicating a lack of recognition by the latter (red and gray lines). Notably, MART-1^G31S^ cells expressing the T-gRNA showed robust killing by T cells, with a cytotoxicity profile similar to MART-1^WT^ cells (Fig. 4C, orange dotted line). Despite only ~10% RNA editing at the G31S site, the resulting partial correction was sufficient to generate a functional immunogenic editope, again underscoring that even modest editing can restore effective T cell recognition (Fig. 4A–C).

We next evaluated this approach in vivo using a melanoma xenograft mouse model. Immunodeficient mice were injected subcutaneously with either MART-1^WT^ or MART-1^G31S^ cells to induce tumor formation. After tumors were established, mice received adoptive T cell therapy (ACT) with MART-1 specific T cells (Fig. EV3F). As expected, tumors derived from MART-1^WT^ cells were completely eliminated following two rounds of ACT, while tumors in untreated controls progressed to the experimental endpoint (Fig. EV3G), validating the working parameters of the system. To assess the effect of RNA editing in this context, we repeated the experiment, but now injected MART-1^G31S^ cells expressing either the NT- or T-SPEAR gRNA into immunodeficient mice (Fig. 4D). Mice injected with MART-1^G31S^ cells expressing the NT-gRNA failed to respond to ACT, and tumors continued to grow. In contrast, tumors derived from MART-1^G31S^ cells expressing the T-gRNA showed marked regression following ACT (Figs. 4E and EV3H). Furthermore, we also observed prolonged survival for mice carrying tumors derived from MART-1^G31S^ cells expressing the T-gRNA relative to those expressing the NT-gRNA (Fig. 4F).

Together, these findings demonstrate that SPEAR gRNAs can successfully reprogram tumor cells by recruiting endogenous ADAR enzymes to generate immunogenic *editopes*, enabling precise T cell targeting and tumor control without affecting target mRNA expression.

### A pipeline for the generation of patient-specific editopes across seven cancer types

Thus far, we have used programmable RNA editing to “generate” a single known immunogenic epitope at the RNA level. To extend our proof-of-concept findings into a broader oncologic context, we employed a pipeline to predict potential neoepitopes that can be rewritten onto tumors to generate immunogenic editopes. To this end, we developed the neoADARgen algorithm, which incorporates patient-specific somatic mutations expressed exclusively in tumor cells, thereby enabling selective targeting of tumor tissue while sparing normal cells (described in detail in Methods). We then computationally applied the algorithm to genomic and transcriptomic data from The Cancer Genome Atlas (TCGA), focusing on seven major cancer types: breast cancer (BRCA), skin melanoma (SKCM), lung adenocarcinoma (LUAD), lung squamous cell carcinoma (LUSC), glioblastoma (GBM), ovarian cancer (OV), and liver hepatocellular carcinoma (LIHC). For each of the 3224 patients in these cohorts, we integrated somatic mutation profiles, HLA haplotype and gene expression data to predict the likelihood that individual mutations could result in neoantigen formation. In total, 808,297 somatic mutations were analyzed, resulting in 19,311 predicted novel neoantigens across all HLA haplotypes. Prior to applying NeoADARgen, patients harbored an average of 4.3 predicted epitopes. Following the algorithm’s application, this number increased to an average of 10.3 epitopes per patient, demonstrating a consistent enhancement in predicted immunogenicity across all cancer types (Table 1).

**Table 1:** Summary of NeoADARgen-predicted neoantigens across seven TCGA cancer cohorts.

TCGA project	Total number of patients included	Epitopes prior to NeoADARgen algorithm (total/avg. per patient)	Patients with novel epitopes identified	Novel epitopes predicted (total/avg. per patient)	Shared novel epitopes
BRCA	965	2431/2.5	801	3675/4.6	39
SKCM	467	3089/6.6	434	4642/10.7	41
OV	289	3323/11.5	273	3190/11.7	6
GBM	147	188/1.3	107	289/2.7	4
LIHC	362	702/1.9	267	1029/3.9	4
LUAD	507	1999/3.9	474	3185/6.7	27
LUSC	487	2029/4.2	466	3301/7.1	15

Application of the NeoADARgen algorithm to 3224 TCGA patients revealed a consistent increase in predicted neoantigens across all analyzed cancer types. The table summarizes cohort sizes, baseline epitope counts, and the number of novel epitopes identified following RNA editing–based prediction.

For instance, in breast cancer (BRCA), patients initially exhibited an average of 2.5 predicted neoantigens. After applying our algorithm, 801 patients were predicted to generate a total of 3675 editopes, corresponding to an average of about 6.8 neoantigens per patient. These editopes could, in principle, be generated by the delivery of multiple SPEAR ADAR-engagers, thereby increasing the number of recognizable antigens and enhancing the likelihood of effective immune targeting in low-TMB tumors.

### Using ADAR-engagers to “write” editopes onto individual tumors, starting from shared neoepitopes

ADAR-engager-based therapies, whether aimed at correcting pathogenic variants or generating personalized editopes, are inherently limited in scalability. To broaden the applicability of our editope-generation approach to a wider patient population, we sought to identify a limited set of shared editopes (and their corresponding SPEAR-gRNAs) that could cover a large fraction of cancer cases. To this end, we analyzed the 50 most frequent somatic mutations documented in the NCI Genomic Data Commons (GDC) database (Grossman et al, 2016) across all cancer types, along with the 50 most common HLA alleles represented in the same database. We then applied our algorithm to evaluate each possible mutation-HLA combination and identify potential editopes predicted to be strong neoantigens.

**Figure 5:**
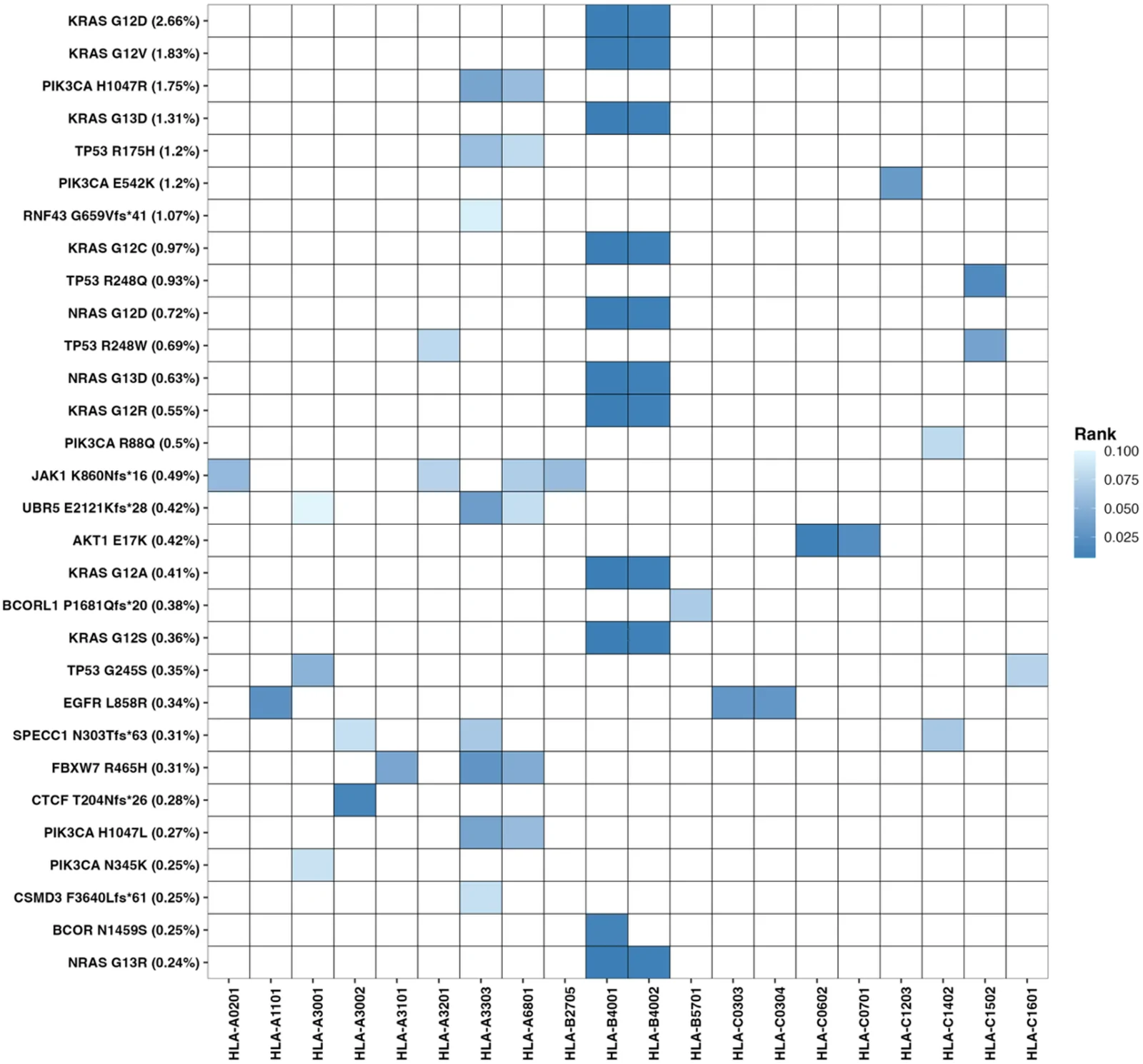
Neoantigens that can be generated next to prevalent hotspot somatic mutations. A heatmap of the 50 most common somatic mutations across all cancer types and the 50 most prevalent HLA types, illustrating all possible combinations that can generate novel potent neoantigens. Only rows and columns in which a result was detected are shown. Source data are available online for this figure.

For each combination, we first used a peptide-HLA-binding prediction tool to calculate the best possible rank before editing (Reynisson et al, 2020). If no high-affinity neoantigen was predicted at a particular site, we ran our algorithm to search for nearby adenosines that could be targeted for editing to generate a neoantigenic peptide. Only combinations predicted to yield a potent neoantigen after editing were retained as viable (Fig. 5). As an illustrative example, KRAS, one of the most frequently mutated oncogenes, harbors mutations at codon 12, where substitution of glycine is a common driver event in pancreatic, colorectal, and lung cancers. These mutations lead to the constitutive activation of the KRAS protein, promoting oncogenic signaling and uncontrolled proliferation^21^. According to the GDC database, KRAS^G12^ mutations occur in ~7% of all cancers.

Focusing on this hotspot, our algorithm identified a candidate editope for four common HLA types by targeting an adenosine located eleven bases downstream of the mutation site. The corresponding mRNA sequence is: CUUGUGGUAGUUGGAGCUG**X**UGGCGUAGGC*AGAGUGCCU, where **X** represents the mutation site (as multiple SNVs at this hotspot are known KRAS mutations), and the asterisk (*) denotes the adenosine predicted for editing. For this target, the SPEAR gRNA sequence would be UUGGAUCAUAUUCGUCCACAUUUAGAUUCUGAAUUAGCUGUAUCGUCAAGGCACUCUCGCCUACGCCA**Y**CAGCU, where the underlined bases are mismatched to the mRNA sequence for editing purposes and **Y** indicates the base complementary to **X**. The resulting neoantigen achieved a predicted rank of 0.01% and a binding affinity of 6.35 nM. Notably, this same editope can also be generated for tumors carrying mutations in KRAS^G13^, as well as NRAS^G12^ or NRAS^G13^ due to gene homology, requiring only minor adjustments to the designed gRNA, potentially extending applicability to an additional 2% of cancers. This example highlights how our approach can exploit recurrent hotspot mutations to generate editopes across multiple patients. Such shared targets could enable the development of off-the-shelf SPEAR gRNAs for tumors with common mutational profiles, substantially increasing the clinical and pharmaceutical utility of the editope generation strategy.

## Discussion

Enhancing the number and immunogenicity of neoantigens within tumors is a critical step for improving the efficacy of immunotherapy. This is especially relevant for cancer vaccines, where the ability to design potent, tumor-specific neoantigens enables the patient to direct an immune response against these engineered targets. However, the selective generation of neoantigens remains challenging.

Here, we introduce a novel approach for neoantigen engineering based on the recruitment of endogenous ADAR1 via SPEAR gRNAs. This technology enables programmable RNA editing through gRNA delivery and without the need for exogenous enzymes. Furthermore, RNA editing, as opposed to DNA editing, enhances safety by reducing the risk of permanent genomic alterations. In our proof-of-concept experiments, we demonstrate that ADAR1-mediated RNA editing can generate known immunogenic neoepitopes at the RNA level and functionally restore T cell recognition and tumor control. Our data further show that targeted RNA editing can modulate the immunogenicity of an epitope by altering its RNA sequence while preserving its translation, processing, and presentation by MHC-I molecules. This RNA-edited epitope, termed editope, can be recognized by and activate antigen-specific T cells, ultimately inducing T cell-mediated cytotoxicity.

After establishing the feasibility of editope generation both in vitro and in vivo, we developed a computational pipeline capable of designing SPEAR gRNAs tailored to each patient’s HLA genotype while leveraging recurrent mutations across tumor populations. This approach aims to ensure that edits are preferentially introduced in tumor cells by anchoring guide RNA binding to somatic mutations, while maximizing the likelihood that the resulting peptides are presented as neoantigens.

Applying our computational algorithm to thousands of tumor samples across seven cancer types, we found that our approach could nearly triple the number of predicted potent neoantigens for the majority of patients. We also showcased several editing candidates derived from prevalent somatic mutations that can serve as entry points for broad clinical applications of oncologic patients. Our approach relies on mutation-specific hybridization, a principle widely used in molecular technologies that can discriminate single-nucleotide differences. With appropriate guide design, this strategy is expected to enable preferential targeting of transcripts harboring tumor-specific somatic mutations, with potentially even greater specificity for larger sequence alterations such as insertions or deletions. However, we note that while such hybridization-based specificity is well established in other systems, the degree to which SPEAR gRNAs can selectively discriminate between mutant and wild-type transcripts in a cellular context remains to be experimentally validated and will be an important aspect of future work.

While our in silico predictions provide a compelling roadmap, we acknowledge the inherent complexity of immune recognition. We relied on a well-established MHC-binding prediction tool that estimates the likelihood of a peptide-MHC interaction based on percentile ranking relative to a large set of naturally occurring peptides (Reynisson et al, 2020). Additionally, we incorporated gene expression levels into our prioritization strategy to increase the biological plausibility of candidate neoepitopes. However, accurate modeling of neoantigen presentation remains challenging, as it depends on multiple cellular processes, including proteasomal cleavage, peptide trimming, transport through the antigen-processing machinery, and peptide loading onto MHC molecules. Computational modeling of these steps remains incomplete and continues to be an active area of research (Gartner et al, 2021). Importantly, predicted neoepitopes identified through HLA-binding algorithms represent candidate peptides and do not necessarily correspond to epitopes that are naturally processed, presented on tumor cells, or capable of eliciting T cell responses; therefore, individual candidates ultimately require experimental validation to confirm their presentation and immunogenicity. More broadly, tumor immune escape mechanisms may further limit the effectiveness of predicted neoantigens. For example, somatic loss of HLA class I heterozygosity is a well-described mechanism by which tumors evade immune recognition (Garrido and Algarra, 2001). This is a risk that is shared with neoepitope vaccines; however, recent data support substantial efficacy for such vaccines (Rojas et al, 2023), suggesting that our platform should theoretically also overcome this concern.

A general limitation of ADAR-engagers is editing efficiency. Previous studies have reported variable editing levels across targets and delivery formats (Reautschnig et al, 2022; Qu et al, 2019; Merkle et al, 2019; Katrekar et al, 2022; Reautschnig et al, 2025; Yi et al, 2022; Byrne et al, 2025; Smargon et al, 2025; Sun et al, 2025). In our context, however, high editing efficiency is not required: the generation of even a small number of edited transcripts appears sufficient to trigger T cell recognition, as we observed both in vitro and in vivo (Figs. 3 and 4). Unlike conventional base editing applications that aim for full protein restoration, our goal is to create peptides that are processed and presented as non-self, enabling immune activation by a relatively small fraction of edited mRNAs, and for this purpose, even low editing efficiency is sufficient. Notably, we observed that gRNA expression levels were substantially lower than those of the target transcript (Fig. EV3E), suggesting that increasing gRNA abundance may represent a potential strategy to further enhance editing efficiency.

An additional limitation of the current study is that gRNA expression was ensured through pre-engineering of tumor cells prior to implantation. While this experimental design enabled a controlled evaluation of the immunological consequences of SPEAR-mediated RNA editing, it does not fully recapitulate a therapeutic scenario in which guide RNAs must be efficiently delivered to established tumors in vivo. Addressing this challenge will be critical for clinical translation. Several delivery strategies could be envisioned, including lipid nanoparticle-based systems, viral vectors, or tumor-targeted RNA delivery platforms, which have shown increasing promise in recent years.

Beyond its use as a standalone approach, SPEAR-mediated RNA editing could be integrated with existing cancer therapies. For instance, combining this strategy with immune checkpoint blockade may enhance T cell responses against edited tumor cells. Similarly, coupling SPEAR with adoptive T cell therapies or cancer vaccines could potentiate the immune response against editopes. More broadly, this approach provides a flexible framework for generating customizable neoepitopes across tumor types, potentially enabling personalized immunotherapeutic strategies.

Overall, the approach presented here demonstrates that harnessing programmable RNA editing via SPEAR gRNAs and endogenous ADAR1 provides a safer, more precise, and adaptable platform for generating tumor-specific neoantigens at the RNA level, which we call editopes. This technology has the potential to enhance immunogenicity in poorly immunogenic tumors and serve as a powerful complement to current immunotherapeutic strategies.

## Methods

**Table:** Reagents and tools table

Reagent/resource	Reference or source	Identifier or catalog number
**Experimental models**		
HEK293T cells	ATCC	Cat# CRL-3216; RRID:CVCL_0063
Jurkat-DMF5 cells	Cetin et al, 2024	
624-mel cells		RRID:CVCL_8054
624-mel MART-1^G31S^ cell line	This study	
Immunodeficient NXG mice	Janvier Laboratory	
**Recombinant DNA**		
pcDNA3.1(-) mCherry-2A-FLAG-eGFP^W58X^	Montiel-González et al, 2016	
pLoxPuro	Arakawa et al, 2001	
pENTR-U6	Montiel-González et al, 2016	
pLV hU6-gRNA hPGK1-BSD	VectorBuilder	VB210525-1302qyy
pLV hU6-gRNA hPGK-MART-1^WT^-T2A or hPGK-MART-1^G31S^-T2A	This study	
pT hU6-gRNA hPGK-eBFP-P2A-BSD	This study	
pPGK-SB13	Addgene	Cat#236078; RRID: Addgene_236078
AALN-BE4max	Addgene	Cat#138161; RRID: Addgene_138161
pCMV-T7-ABEmax(7.10)-SpCas9-NG-P2A-EGFP	Addgene	Cat#140005; RRID: Addgene_140005
pFYF1320 EGFP Site#1	Addgene	Cat#47511; RRID: Addgene_47511
**Antibodies**		
PE anti-human CD69	BioLegend	Cat# 310906
APC anti-human CD2	BioLegend	Cat# 300214
**Oligonucleotides and other sequence-based reagents**		
All oligonucleotides	This study	Appendix Table S1
**Chemicals, enzymes and other reagents**		
Rapid DNA Ligation kit	Merck	Cat# 11635379001
Q5® High-Fidelity DNA Polymerase	NEB	Cat# M0491
NEBuilder® HiFi DNA Assembly Master Mix	NEB	Cat# E2621
SpeI-HF	NEB	Cat# R3133
NsiI-HF	NEB	Cat# R3127
XhoI	NEB	Cat# R0146
AgeI-HF	NEB	Cat# R3552
QuickExtract™ DNA Extraction Kit	Lucigen	Cat #QE0905T
7AAD	Invitrogen	Cat# A1310
TURBO DNA-*free*™ Kit	Invitrogen	Cat# AM1907
One-step RT-PCR kit	Qiagen	Cat# 210212
iScript Select cDNA synthesis kit	Bio-Rad	Cat# 1708896
SsoAdvanced Universal SYBR Green Supermix	Bio-Rad	Cat# 1725270
TRIzol™ Reagent	Invitrogen	Cat# 15596018
Matrigel Basement Membrane Matrix	Corning	Cat# 356234
**Software**		
MultiEditR	Kluesner et al, 2021; https://moriaritylab.shinyapps.io/multieditr/	
CRISPResso2	Clement et al, 2019; https://crispresso2.pinellolab.org/submission	
**Other**		
xCELLigence	Agilent	

### Plasmids

#### pcDNA3.1(-) mCherry-P2A-eGFP^W58X^ puro

This plasmid was generated by inserting a puromycin resistance cassette into the pcDNA3.1(−) mCherry-2A-FLAG-eGFP^W58X^ backbone, which was linearized with BglII. The parental plasmid was a kind gift from Dr. Joshua Rosenthal (University of Chicago) and previously described in *REF* (Montiel-González et al, 2016). The puromycin selection cassette was excised from plasmid pLoxPuro (Arakawa et al, 2001) via BamHI digestion and ligated into the linearized vector using the Rapid DNA Ligation kit (Merck, Cat# 11635379001).

#### Plasmids encoding ADAR1-recruiting gRNAs for eGFP^W58X^

Guide RNA-encoding DNA fragments were generated by inserting single-stranded DNA (ssDNA) oligonucleotides into a pENTR-U6 backbone (a kind gift of Dr. Joshua Rosenthal, University of Chicago), which was linearized via PCR using Q5® High-Fidelity DNA Polymerase (NEB, Cat# M0491) and primers #1 and #2 (see Appendix Table S1). One or two ssDNA oligos or a dsDNA fragment were inserted into the PCR-amplified vector using NEBuilder® HiFi DNA Assembly Master Mix (NEB, Cat# E2621). All ssDNA oligonucleotides were ordered by Sigma-Aldrich, and the dsDNA fragments by Integrated DNA Technologies (IDT). Nucleotide sequences are provided below, with NEBuilder-compatible overlapping regions shown in lowercase. All plasmids were verified by Sanger sequencing. Detailed plasmid maps are available in the Appendix as text files.

The following gRNA-expressing plasmids were generated for eGFP^W58X^ (Fig. EV1B,C):

- **pENTR-U6 GluRv1-16** (eGFP^W58X^) – inserted ssDNA oligo: tatatcttgtggaaaggacgaaacaccGGTGAATAGTATAACAATATGCTAAATGTTGTTATAGTATCCACCGTTGGCCATGGAACAGttttttctagacccagctttcttgtac
- **pENTR-U6 GluRv4-16** (eGFP^W58X^) – inserted ssDNA oligo: tatatcttgtggaaaggacgaaacaccGGTGAAGAGGAGAACAATATGCTAAATGTTGTTCTCGTCTCCACCGTTGGCCATGGAACAGttttttctagacccagctttcttgtac
- **pENTR-U6 GluRv9.4-16** (eGFP^W58X^) – inserted ssDNA oligo: tcttgtggaaaggacgaaacaccGGTGTCGAGAAGAGGAGAACAATATGCTAAATGTTGTTCTCGTCTCCTCGACACCGTTGGCCATGGAACAGttttttctagacccagctttctt
- **pENTR-U6 GluRv9.4-38** (eGFP^W58X^) – inserted ssDNA oligos: (1) tcttgtggaaaggacgaaacaccGGTGTCGAGAAGAGGAGAACAATATGCTAAATGTTGTTCTCGTCTCCTCGACACCGTATGTCAGGGTAGTGACAA; (2) acaagaaagctgggtctagaaaaaaCTGTTCCATGGCCAACACTTGTCACTACCCTGACATACGGTGTCGAGGAGACGAGAACAACAT
- **pENTR-U6 LEAPER-70** (eGFP^W58X^) – inserted ssDNA oligo: tcttgtggaaaggacgaaacaccGGATTGCACGCCGTATGTCAGGGTAGTGACAAGTGTTGGCCATGGAACAGGTAGTTTTCCAGTAGTGAAATttttttctagacccagctttctt
- **pENTR-U6 LEAPER-151 (**Qu et al, 2019**)** (eGFP^W58X^) – inserted ssDNA oligos: (1) tatcttgtggaaaggacgaaacaccGGTCGTGCTGTTTCATGTGGTCGGGGTACCTGCTGAAGCATTGCACGCCGTATGTCAGGGTAGTGACAAGTGTTGGCCATGGAACAGGTAGTTTT; (2) acaagaaagctgggtctagaaa aaaAAGGAGAGGGCGATGCCACCTATGGCAAGCTGACCCTGAAGTTTATTTGCACTACTGGAAAACTACCTGTTCCATGGCCAACACTTGTCACTACC
- **pENTR-U6 LEAPER-151 (38-4ntMM-33-C-75;** Uzonyi et al, 2021**)** (eGFP^W58X^) – inserted ssDNA oligos: (1) tatcttgtggaaaggacgaaacaccGGTCGTGCTGTTTCATGTGGTCGGGGTACCTGCTGAAGCAAACGACGCCGTATGTCAGGGTAGTGACAAGTGTTGGCCATGGAACAGGTAGTTTT; (2) acaagaaagctgggtctagaaaaaaAAGGAGAGGGCGATGCCACCTATGGCAAGCTGACCCTGAAGTTTATTTGCACTACTGGAAAACTACCTGTTCCATGGCCAACACTTGTCACTACC
- **pENTR-U6 38-4ntMM-33-C-31 (sdl 107)** (eGFP^W58X^) – inserted ssDNA oligos: (1) tatcttgtggaaaggacgaaacaccGGTCGTGCTGTTTCATGTGGTCGGGGTACCTGCTGAAGCAAACGACGCCGTATGTCAGGGTAGTGACAAGTGTTGGCCATGGAACAGGTAGTTTT; (2) acaagaaagctgggtctagaa aaaaATTTGCACTACTGGAAAACTACCTGTTCCATGGCCAACACTTGTCACTACC
- **pENTR-U6 38-4ntMM-33-C-16 (sdl 92)** (eGFP^W58X^) – inserted ssDNA oligos: (1) tatcttgtggaaaggacgaaacaccGGTCGTGCTGTTTCATGTGGTCGGGGTACCTGCTGAAGCAAACGACGCCGTATGTCAGGGTAGTGACAAGTGTTGGCCATGGAACAGGTAGTTTT; (2) acaagaaagctgggtctagaaa aaaAAACTACCTGTTCCATGGCCAACACTTGTCACTACC
- **pENTR-U6 20-4ntMM-33-C-75 (sdl 133)** (eGFP^W58X^) – inserted ssDNA oligos: (1) tatcttgtggaaaggacgaaacaccGGTCGGGGTACCTGCTGAAGCAAACGACGCCGTATGTCAGGGTAGTGACAAGTGTTGGCCATGGAACAGGTAGTTTT; (2) acaagaaagctgggtctagaaaaaaAAGGAGAGGGCG ATGCCACCTATGGCAAGCTGACCCTGAAGTTTATTTGCACTACTGGAAAACTACCTGTTCCATGGCCAACACTTGTCACTACC
- **pENTR-U6 10-4ntMM-33-C-75 (sdl 133)** (eGFP^W58X^) – inserted ssDNA oligos: (1) tatcttgtggaaaggacgaaacaccGGTGCTGAAGCAAACGACGCCGTATGTCAGGGTAGTGACAAGTGTTGGCCATGGAACAGGTAGTTTT; (2) acaagaaagctgggtctagaaaaaaAAGGAGAGGGCGATGCCACCTAT GGCAAGCTGACCCTGAAGTTTATTTGCACTACTGGAAAACTACCTGTTCCATGGCCAACACTTGTCACTACC
- **pENTR-U6 20-4ntMM-33-C-16 (sdl 74, SPEAR)** (eGFP^W58X^) – inserted ssDNA oligo: cttgtggaaaggacgaaacaccGGTCGGGGTACCTGCTGAAGCAAACGACGCCGTATGTCAGGGTAGTGACAAGTGTTGGCCATGGAACAGGTAGTTTttttttctagacccagctttct

### Plasmids for in vitro T cell activation assay

The plasmids used for the proof-of-concept T cell activation assay (Figs. 3 and EV2) contain a dual-expression cassette structure: a hU6-driven gRNA expression cassette, followed by a hPGK1-driven cassette encoding a codon-optimized MART-1 (coMART-1), eBFP, and the blasticidin resistance gene (BSD), separated by two different self-cleaving 2 A peptides (Liu et al, 2017) (Fig. EV2B). This design enables both antibiotic selection and monitoring of MART-1 expression via eBFP fluorescence. We began by ordering a custom plasmid backbone from VectorBuilder containing the hU6-gRNA and hPGK1-BSD expression cassettes (VectorBuilder ID: VB210525-1302qyy; this ID can be used to retrieve detailed information about the vector on vectorbuilder.com). The vector was linearized using SpeI-HF (NEB, Cat# R3133) and BsrGI-HF (NEB, Cat# R3575). The individual components, hU6-gRNA cassette, hPGK-MART-1^WT^-T2A or hPGK-MART-1^G31S^-T2A, and eBFP-P2A-BSD, were amplified separately by PCR using Q5® High-Fidelity DNA Polymerase (NEB, Cat# M0491) from existing plasmids and assembled into the linearized backbone using NEBuilder® HiFi DNA Assembly Master Mix (NEB, Cat# E2621). The resulting plasmids express either a non-targeting (NT) or targeting (T) SPEAR gRNA as follows:

- **NT-SPEAR gRNA** (control): TCGGGGTACCTGCTGAAGCAAACGACGCCGTATGTCAGGGTAGTGACAAGTGTTGGCCATGGAACAGGTAGTTT
- **T-SPEAR gRNA** (coMART-1^G31S^): TCCGGCAGTACCAGCAGCCGTAGTGCAGCAGCACGCCTAGGATCACTGTCAGGAGGCCGATCCCAGCAAGCTCC

### Plasmids for the generation of known immunogenic neoepitopes, in vivo T cell activation and tumor elimination assay

The plasmids used for the generation of known immunogenic neoepitopes and the in vivo experiments (Figs. 2, 4 and EV3) were also designed with a dual-expression cassette structure comprising a hU6-driven gRNA expression cassette, followed by an hPGK1-driven cassette encoding eBFP and the blasticidin resistance gene (BSD), separated by a self-cleaving 2A peptide (Fig. EV3B). This configuration enables antibiotic selection and allows monitoring of plasmid integration through eBFP fluorescence. To enable stable genomic integration, the dual-expression cassette was cloned into a pT vector containing the necessary inverted terminal repeats (ITRs) for Sleeping Beauty transposon-mediated integration (Cui et al, 2002). The vector was linearized using NsiI-HF (NEB, Cat# R3127) and XhoI (NEB, Cat# R0146). The individual elements, hU6-gRNA cassette and hPGK-eBFP-P2A-BSD, were amplified separately by PCR using Q5® High-Fidelity DNA Polymerase (NEB, Cat# M0491) from existing plasmids and assembled into the linearized backbone using NEBuilder® HiFi DNA Assembly Master Mix (NEB, Cat# E2621). The resulting plasmids express either a non-targeting (NT) or targeting (T) SPEAR gRNA as follows:

- **NT-SPEAR gRNA** (control): TCGGGGTACCTGCTGAAGCAAACGACGCCGTATGTCAGGGTAGTGACAAGTGTTGGCCATGGAACAGGTAGTTT
- **T-SPEAR gRNA** (TP53^Y220C^): TTACACATGTAGTTGTAGTGCTACGTGGTACAGTCAGAGCCAACCTCAGGCGGCTCACAGGGCACCACCACACT
- **T-SPEAR gRNA** (USP9X^Y2009C^): GGAGGAGGGTTTAAGTAAACCGGTTTACATGTAAGCAGTTTTTTCATAAACTGAAAACACTCCATACTGTACTG
- **T-SPEAR gRNA** (C6orf89^Y813C^): ACCAAAGGCTGGACCCCTCTCCGAATAACGTAGACCTTCCAGTGGGTGGTGTCGACACAGCCGATATCCCCTGG
- **T-SPEAR gRNA** (MART-1^G31S^): TTCTACAATACCAACAGCCGTACTGCAGTAAGACTCCCAGGATCACTGTCAGGATGCCGATCCCAGCGGCCTCT

In the in vivo experiments, to ensure stable integration, these plasmids were co-transfected with pPGK-SB13, a Sleeping Beauty transposase expression plasmid. This plasmid was a gift from Masashi Narita (Addgene plasmid #236078; http://n2t.net/addgene:236078; RRID: Addgene_236078) (Chan et al, 2024).

### Plasmids for genome base editing and single guide RNA (sgRNA) expression

The plasmid AALN-BE4max was a gift from David Liu (Addgene plasmid #138161; http://n2t.net/addgene:138161; RRID: Addgene_138161) and encodes a modified cytosine base editor consisting of rAPOBEC1(AALN)-nSpCas9-UGI-UGI, in which APOBEC1 has been engineered to reduce off-target activity (Doman et al, 2020). To facilitate the identification of transfected cells, we inserted an eGFP reporter into this plasmid. Specifically, plasmid AALN-BE4max was digested with AgeI-HF (NEB, Cat# R3552) to linearize it between the UGI-SV40_NLS and 6xHis tag regions. The insert, encoding P2A-EGFP-SV40_NLS-6xHis, was PCR-amplified from pCMV-T7-ABEmax(7.10)-SpCas9-NG-P2A-eGFP (Addgene plasmid #140005; gift from Benjamin Kleinstiver; http://n2t.net/addgene:140005; RRID: Addgene_140005) (Walton et al, 2020) using Q5® High-Fidelity DNA Polymerase (NEB, Cat# M0491) and primers #3 and #4 (see Appendix Table S1). The insert was assembled into the linearized backbone using NEBuilder® HiFi DNA Assembly Master Mix (NEB, Cat# E5520).

For sgRNA expression, we used the vector pFYF1320 eGFP Site#1, a gift from Keith Joung (Addgene plasmid #47511; http://n2t.net/addgene:47511; RRID: Addgene_47511) (Fu et al, 2013), which originally encoded a sgRNA targeting eGFP. This sequence was replaced with an sgRNA targeting the *MART-1* gene as follows. The backbone was amplified using Q5® PCR to exclude the eGFP-targeting region using primers #5 and #6 (see Appendix Table S1). A ssDNA oligo containing the protospacer for the *MART-1*-targeting sgRNA (atatatcttgtggaaaggacgaaacaccggGATGCCGATC CCAGCGGCCCgttttagagctagaaatagcaagtt) was assembled into the linearized backbone using NEBuilder® HiFi DNA Assembly Master Mix (NEB, Cat# E5520).

### DNA oligonucleotides

All primers used in this study can be found in (Appendix Table S1) and were designed using Primer-BLAST (Ye et al, 2012), AmplifX 2.1.1 (by Nicolas Jullien; Aix-Marseille Univ, CNRS, INP, Inst Neurophysiopathol, Marseille, France - https://inp.univ-amu.fr/en/amplifx-manage-test-and-design-your-primers-for-pcr), or ApE (Davis and Jorgensen, 2022).

### Cell culture and cell lines generation

HEK293T cells (ATCC, Cat# CRL-3216, RRID: CVCL_0063) were cultured at 37 °C in a humidified incubator with 5% CO_2_ in high-glucose DMEM (Sigma-Aldrich, Cat# D6429), supplemented with 10% FBS (Thermo Fisher, Cat# 10270106) and 1% penicillin/streptomycin (Sigma-Aldrich, Cat#P4333).

The stable dual-reporter cell line expressing mCherry-P2A-eGFP^W58X^-puro was generated by transfecting the plasmid into HEK293T cells using Lipofectamine 2000 (Thermo Fisher, Cat# 11668019) according to the manufacturer’s protocol. 48 h after transfection, cells were plated by limiting dilution into 96-well plates in medium supplemented with puromycin (1.5 µg/ml). Clonal populations were identified by microscopic inspection, and single clones were screened via flow cytometry analysis to select a single clone stably expressing the dual fluorescence reporter plasmid with no detectable eGFP background.

HEK293T cells stably expressing the constructs for the in vitro T cell activation assay (Figs. 3 and EV2), which included hU6-gRNA (T- or NT-) and hPGK1-coMART-1^WT or G31S^-2A-eBFP-2A-BSD were generated similarly. Cells were transfected using Lipofectamine 2000, expanded for 48 h, and then selected with blasticidin (15 µg/ml). As soon as non-transfected cells died, eBFP⁺ cells were sorted by flow cytometry. The expression of MART-1^WT or G31S^ and the presence of SPEAR-mediated editing were confirmed by RT-PCR and Sanger sequencing.

Jurkat cells expressing the DMF5 TCR were cultured at 37 °C and 5% CO_2_ in RPMI 1640 (Sigma-Aldrich, Cat# R8758) supplemented with 10% FBS (Thermo Fisher, Cat# 10270106), 2 mM L-glutamine (Gibco, Cat# 25030-024) and 1X penicillin/streptomycin (Sigma-Aldrich, Cat#P4333). Jurkat-DMF5 cells were generated as previously described (Cetin et al, 2024) using a lentivirally encoded, single-ORF TCR sequence separated by a furin-T2A cleavage sequence, followed by 12 days of puromycin selection for the transgenic TCR.

624-mel cells (RRID:CVCL_8054) were cultured at 37 °C and 5% CO_2_ in high-glucose DMEM (Sigma-Aldrich, Cat# D6429) supplemented with 10% FBS (Thermo Fisher, Cat# 10270106) and 1% penicillin/streptomycin (Sigma-Aldrich, Cat#P4333).

The 624-mel MART-1^G31S^ cell line was generated via DNA base editing (see below). For in vivo experiments (Figs. 4 and EV3), 624-mel MART-1^G31S^ cells were transfected with plasmids expressing either a T- or NT-gRNA together with pPGK-SB13 (Sleeping Beauty transposase) in a 2:1 ratio, using Lipofectamine 2000 (Thermo Fisher, Cat# 11668019). After 48 h, cells were expanded and selected with blasticidin (5 µg/ml). Once selection was complete, BFP⁺ cells were sorted by flow cytometry. Successful expression of MART-1^G31S^ and gRNA-dependent RNA editing were confirmed by RT-PCR and Sanger sequencing.

All cell lines were authenticated using Multiplex Cell Authentication by Multiplexion (Heidelberg, Germany) as described in *REF* (Castro et al, 2013). Additionally, the purity of cell lines was validated using the Multiplex cell Contamination Test by Multiplexion (Heidelberg, Germany) as described in *REF* (Schmitt and Pawlita, 2009). No Mycoplasma, SMRV or interspecies contamination was detected.

### Transient transfection of gRNA-encoding plasmids

For transient gRNA expression experiments using the eGFP^W58X^ dual-reporter system (Fig. 1) and for the generation of known immunogenic neoepitopes at endogenous target sites (Fig. 2), cells were seeded in 24-well plates at a density of 1.5 × 10^5^ cells per well one day before transfection. Cells were transfected with 140 fmol of the indicated gRNA-encoding plasmid using Lipofectamine 2000 (Thermo Fisher, Cat# 11668019), according to the manufacturer’s instructions. For the eGFP^W58X^ reporter experiments, dual-reporter HEK293T cells were transfected with plasmids encoding the indicated gRNAs targeting eGFP^W58X^ and harvested 48 h after transfection for flow cytometry and/or RNA extraction.

For endogenous *editope*-generation experiments, HEK293T or 624-mel cells were transfected with plasmids encoding targeting (T) or non-targeting (NT) SPEAR gRNAs directed against the indicated endogenous target sites. Forty-eight hours after transfection, eBFP-positive cells were sorted by flow cytometry before RNA extraction. RNA was then analyzed by RT-PCR, Sanger sequencing, and editing quantification using MultiEditR (Kluesner et al, 2021).

### Genome base editing with a cytosine base editor (CBE)

10^5^ 624-mel cells were seeded in 500 μl complete growth medium (DMEM, FBS 10%, P/S 1%) in a flat-bottom 24-well plate. After 18–24 h, the complete growth medium was replaced with 250 μl antibiotic-free reduced serum medium (OptiMEM, Gibco, Cat #31985062). Cells were transfected with a mixture of 2 μl lipofectamine 2000, 500 ng sgRNA vector and 1 μg base editor vector diluted in 100 μl OptiMEM following the manufacturer’s instructions. After 6 h, the transfection medium was replaced with 500 μl complete growth medium. After 72 h, the medium was removed, and cells were washed with 250 μl PBS. To detach the cells, 100 μl trypsin were added, and cells were incubated for 1 min at 37 °C. Trypsin was inactivated by adding 400 μl prewarmed complete growth medium. Detached cells were filtered and transferred to a 5 ml round-bottom polystyrene test tube with a cell strainer snap cap (Corning, Cat #352235). GFP^+^ cells were single-cell sorted into flat-bottom 96-well plates. Single-cell clones were incubated for at least 2 weeks at 37 °C until they were ready to be screened. When ready, medium was removed, and clones were washed once with 100 µl PBS. Cells were detached by adding 20 µl trypsin and resuspended in 200 µl complete growth medium. About 100 µl of each clone were transferred into a new flat-bottom 96-well plate already prepared with 100 µl medium per well to keep and further expand the selected clones. The remaining 100 µl were transferred into another flat-bottom 96-well plate already prepared with 100 µl medium per well for screening the next day. DNA was extracted with QuickExtract™ DNA Extraction Kit (Lucigen, Cat #QE0905T). Briefly, medium was removed from the screening plate, and cells were washed once with 100 µl PBS. 100 µl Quick-Extract DNA solution were added, cells were detached by thoroughly pipetting up and down and transferred into a 96-well PCR plate (or PCR strips). Cells were incubated at 65 °C for 15 min in a thermal cycler, vortexed for 15 s, and incubated at 98 °C for 5 min in a thermal cycler. The extracted DNA was used for PCR and then stored at −80 °C. PCR was performed on the extracted DNA with Q5 High-Fidelity DNA Polymerase from NEB (#M0491S) according to the manufacturer’s instructions and the following conditions. About 5–20 ng of DNA (2.5 μl) were added to 31 μl of RNase-free water, 10 μl of Q5 Buffer, 1 μl of dNTP mix (10 mM each), 2.5 μl of each primer (10 μM), and 0.5 μl of Q5 polymerase in a final reaction volume of 50 μl. *MART-1* amplification was obtained using primers #7 and #8 (Appendix Table S1). The PCR program was run as follows: denaturation at 98 °C for 30 s, followed by 35 cycles of denaturation at 98 °C for 10 s, annealing at 65 °C for 15 s and elongation at 72 °C for 10 s. In the end, a final elongation step at 72 °C was run for 2 min. The correct size of the PCR products (245 bp) was checked on a 1.5% agarose gel. About 5 μl of each product diluted with 10 μl nuclease-free water was sent for Sanger sequencing to Eurofins Genomics Germany GmbH.

### Coculture and related flow cytometry analysis

In the coculture experiment using HEK293T overexpressing either MART-1 or MART-1^G31S^ (Figs. 3 and EV2), we used the following antibodies: PE anti-human CD69 (BioLegend, Cat# 310906) and APC anti-human CD2 (BioLegend, Cat# 300214). We additionally assessed the viability of cells using 7AAD staining (Invitrogen, Cat# A1310). About 2.5 × 10^4^ HEK293T stably expressing either MART-1 or MART-1^G31S^ and the SPEAR gRNAs (targeting or not-targeting MART-1^G31S^) were seeded in a 96-well flat-bottom plate in 40 μl of supplemented DMEM. Twenty-four hours post seeding, 1 × 10^5^ DMF5^+^ Jurkat cells in 35 μl of supplemented RPMI were added to the same wells for a total volume of 75 μl per well. The cells were maintained in coculture for 16 h at 37 °C and 5% CO_2_. Samples were washed twice in FACS buffer (2 mM EDTA and 2% FBS in PBS) and cells were stained using 50 μl of FACS buffer containing the antibodies diluted 1:200. After 20 min of incubation in ice, samples were washed twice and resuspended in a final volume of 200 μl of FACS buffer. Flow cytometer acquisition was done using a BD Canto II (BD Biosciences), and data were analyzed with FlowJo.

### Immunoprecipitation of the HLA class I-peptide complexes

The immunoprecipitation protocol is based on a method we previously published (Gomez-Zepeda et al, 2026). In brief, CNBr-activated Sepharose™ 4B (Cytiva) beads coupled to W6/32 antibody (Leinco Technologies) were prepared as follows. Beads (24 mg per sample) were resuspended in 1 mM HCl and mixed on a rotator for 30 min. Beads were centrifuged at 20×*g* for 4 min at room temperature, and the supernatant was discarded. Coupling buffer (0.5 M NaCl, 0.1 M NaHCO₃, pH 8.3) and W6/32 antibody (0.6 mg per sample) were added, followed by incubation on a rotator for 2 h. Beads were centrifuged as above, and the supernatant was discarded. 0.2 M Glycine solution was added, and samples were incubated on a rotator for 1 h to block remaining active sites. Beads were washed twice with 1× PBS by gentle mixing and centrifugation. Finally, beads were resuspended in 1× PBS, distributed into tubes, and centrifuged immediately prior to lysate addition. Approximately 1 × 10⁸ HEK293T cells (three biological replicates per condition) were lysed by thawing on ice in 750–1000 µL of 2× solubilization buffer (SB; 20 mM CHAPS in 1× PBS supplemented with cOmplete EDTA-free protease inhibitor) on a rocker for 1 h. An additional 750 µL of 1× SB (prepared by diluting 2× SB 1:1 with PBS) was added, followed by incubation on ice with rocking for 1 h. Cells were further lysed using a Diagenode Bioruptor (15 cycles, 30 s on/off, high power, 4 °C). Lysates were incubated on ice with gentle agitation for 30 min and centrifuged at 17,000×*g* for 20 min at 4 °C. Supernatants were transferred to fresh tubes and centrifuged again under the same conditions. The clarified lysates were incubated overnight at 4 °C with antibody-coupled beads on a rotator. The following day, beads were centrifuged at 100×*g* for 4 min at 4 °C, and the supernatant was discarded. Beads were washed twice with 1× PBS and once with LC–MS-grade water, with each wash consisting of incubation on ice with gentle mixing for 1 h followed by centrifugation. Elution of MHC–peptide complexes was performed by adding 15 µL of 10% trifluoroacetic acid (TFA) per mg of beads together with 200 µL of 0.2% TFA. After centrifugation, the supernatant was collected. This was followed by six additional elution steps, each using 200 µL of 0.2% TFA. Eluates per sample were pooled and stored overnight at 4 °C. Peptide purification was performed using Vivacon 500 filters (10 kDa MWCO) (Sartorius). Filters were prewashed twice with 300 µL of 1% TFA by centrifugation at 16,300×*g* for 20 min at 4 °C. Samples were then centrifuged at 16,300×*g* for 10 min at 4 °C, and the supernatants were loaded onto the filters. Filtrates containing peptides were collected. Desalting was performed using an Oasis HLB 96-well µElution plate (Waters). The plate was conditioned with 100% acetonitrile (ACN), equilibrated with 0.1% TFA, and samples were loaded. Columns were washed with 0.1% TFA followed by LC–MS-grade water. Peptides were eluted with 35% ACN containing 0.1% TFA, dried in a vacuum centrifuge concentrator, and stored at −80 °C.

Synthetic peptide and calibration curve preparation: Light and stable isotope heavy-labeled peptide standards (ELAGIGILTV and ELAGIGIL[+7]TV) (JPT Peptide Technologies GmbH, Germany) were reconstituted in 0.1% formic acid (FA). About 100 fmol/µL of light and heavy peptide was used for the identification of the peptides and gradient optimization. Light peptide concentrations of 2.5, 5, 7.5, and 10 fmol/µL were prepared, each containing 5 fmol/µL of the heavy peptide for calibration curve generation. Lyophilized MHC-I peptide samples were reconstituted in 0.1% FA containing the heavy peptide at 5 fmol/µL.

LC–MS analysis of HLA-I peptides: Chromatographic separation was performed using a NanoElute2 system (Bruker) equipped with an Aurora Ultimate™ 25 × 75 mm C18 UHPLC column (IonOpticks), coupled online to a timsTOF Pro 2 mass spectrometer (Bruker). Mobile phases consisted of eluent A (0.1% FA in water) and eluent B (0.1% FA in ACN). Peptides were separated over a 47 min gradient: 2–25% B over 23 min, 25–60% B over 15.3 min, increased to 95% B over 3.8 min, followed by 4.9 min at 95% B. The flow rate was maintained at 400 nL/min. Default loading and equilibration volumes were used. Ionization was performed using a CaptiveSpray source with a capillary voltage of 1600 V, dry gas flow of 3.0 L/min, and dry temperature of 180 °C. To create the PRM method, standard peptide data were acquired by DDA-PASEF mode (with an optimized tims isolation filter that includes +1 precursors (Gomez-Zepeda et al, 2024)). Then, calibration curve and samples data were acquired in PRM-PASEF mode (Brzhozovskiy et al, 2022) using a predefined list of precursor m/z and ion mobility values (provided as source data for Fig. 3D). Three injection replicates were analyzed per sample.

LC–MS data analysis: Raw data from synthetic peptides were analyzed using FragPipe (Yu et al, 2023) (v23.1, including MSFragger v4.3) against a database containing the target peptide sequence and common contaminants (cRAP; 122 sequences). The default “nonspecific-HLA” workflow was used, with a delta mass of +7.017 Da on leucine specified as a variable modification. Peptides were filtered at a 1% false discovery rate (FDR) using strict parsimony. Absolute quantification was performed using Skyline (Pino et al, 2020) (v26.1). FragPipe results were imported to generate a spectral library. Six fragment ions per precursor were selected for quantification (provided as source data for Fig. 3D). Ion mobility filtering was applied using a fixed window of 0.1. Calibration curves were generated using a linear regression through zero (1/(x*x) weighting) based on light-to-heavy ratios at the MS2 level. Peptide concentrations (fmol/µL) were calculated accordingly. Injection replicates were averaged to obtain final sample concentrations.

### Adenosine-to-inosine (A-to-I) editing sites quantification

For A-to-I editing site quantification, RNA was extracted using the RNeasy Mini Kit (Qiagen, Cat# 74106) or Micro Kit (Qiagen, Cat# 74004), depending on cell numbers, and treated with DNase (Invitrogen, Cat# AM1907). One-step RT-PCRs were performed using the One-step RT-PCR kit (Qiagen, Cat# 210212) using primers listed in Appendix Table S1. Additionally, when needed, the same PCR was performed on genomic DNA (extracted using the All Prep DNA/RNA Kit, Qiagen, Cat# 80204) with Q5 High-Fidelity DNA Polymerase (NEB, Cat# M0491) as a no-editing control. All the PCR products were purified (Macherey-Nagel, Cat# 740609) and analyzed by Sanger sequencing. Quantification of editing was performed directly from the Sanger traces using MultiEditR (Kluesner et al, 2021).

### On-target and bystander off-target editing analysis via amplicon-seq

To evaluate in detail on-target and bystander off-target editing on the eGFP W58X reporter, a second PCR with primers containing adapter sequences (#15-16, Appendix Table S1) for MiSeq amplicon sequencing (Eurofins Genomics, GATC services, Germany) was performed using Q5® High-Fidelity DNA Polymerase (NEB, Cat# M0491). Before performing the second PCR, the amplicons from three independent replicates were mixed in a 1:1:1 ratio. Analysis of the amplicon-seq data was performed using CRISPResso2 (Clement et al, 2019).

### RT-qPCR

For the ADAR1, ADAR2 and MLANA gene expression, RNA was extracted using the RNeasy Plus Mini kit (Qiagen, Cat# 74134). Before qPCR, RNA was additionally treated with DNase (Invitrogen, Cat# AM1907). cDNA synthesis was then performed using the iScript Select cDNA synthesis kit (Bio-Rad, Cat# 1708896) with 200 ng of DNAse-digested RNA, oligo-dT and random hexamer primers, according to the manufacturer’s protocol. About 5 ng of cDNA (2.5 ng/μl) were used to set up a 10 μl qPCR reaction using SsoAdvanced Universal SYBR Green Supermix (Bio-Rad, Cat# 1725270). To quantify gRNA expression, RNA extraction was performed using the TRIzol™ Reagent (Invitrogen, Cat# 15596018), following the manufacturer’s instructions. Rigorous DNase treatment was done using the TURBO DNA-*free*™ Kit (Invitrogen, Cat# AM1907). cDNA synthesis and qPCR reactions were performed as described above. Primer efficiency was calculated as following the MIQE guidelines (Bustin et al, 2009). For details about primer efficiency analysis, see Appendix Methods and Appendix Fig. S3. Samples, no-reverse transcriptase (NRT) and non-template controls (NTC) were measured in two technical replicates. Finally, relative expression was calculated using the comparative CT method (ΔΔCT) (Livak and Schmittgen, 2001), normalized to the housekeeping gene PGK1. All primers used for this assay are listed in Appendix Table S1.

### xCELLigence cytotoxicity assay

MART-1 ELAGIGILTV/A2 TCR (DMF5) was transduced into human PBMC. DMF5 positive cells were expanded in the presence of PBMCs feeder cells loaded with MART-1 peptide (ProImmune) and IL2 (70 IU/ml) for 14 days. >90% of T cells were DMF5 positive after the procedure. Cell-mediated cytotoxicity was analyzed in an impedance-based label-free cytotoxicity assay (xCELLigence) system (Peper et al, 2014). About 2 × 10^4^ 624-mel cells (each cell line in three technical replicates; three biological replicates of the MART-1^G31S^ cells expressing either the NT- or T-SPEAR gRNA were compared) were seeded in 50 μl of complete growth medium (DMEM FBS 10% P/S 1%) in a 96-well E-plate (E-Plate 96 PET, Agilent). The attachment of the cells changes the electrical impedance of these electrodes, which is monitored as an increase of the dimensionless “cell index”. After 24 h, 4 × 10^4^ MART-1 specific T cells were added in 50 μl of complete growth medium in half of the samples (condition “+ T cells”), while the other half only received 50 μl of fresh complete growth medium. Lysis of the tumor cells by the T cells causes their detachment from the electrodes, which changes the impedance of the wells, resulting in a decrease in the cell index. The tumor cell growth was continuously monitored from the seeding of the tumor cells until 48 h after the addition of the T cells. The coculture of tumor cells and T cells was incubated for 48 h at 37 °C, 5% CO_2_, and 95% humidity.

### Mice

Immunodeficient NXG mice came from the Janvier Laboratory and were bred at the DKFZ animal facility. 10- to 16-week-old mice were assigned to age-matched experimental groups. Mice were housed under Specific and Opportunistic Pathogen Free (SOPF) conditions and a 12-h day/night cycle. Animal procedures were performed in accordance with all relevant ethical regulations for animal testing and research and approved by the governmental authorities (Regional Administrative Authority Karlsruhe, Germany; permit number 35-9185.81/G-11/22).

### In vivo tumor growth and adoptive T cell therapy (ACT)

On day 1, 500.000 melanoma cells in 100 μl were mixed in a 1:1 volume with 100 μl Matrigel Basement Membrane Matrix (Corning, Cat# 356234) and kept strictly on ice till injection in mice. The cell-Matrigel mix was subcutaneously injected on the right flank of NXG mice. Each cell line was injected into 12 mice. Tumor size was measured every other day starting on day 6. On days 12 and 19, 5 × 10^6^ MART-1 specific T cells in 50 μl PBS were administered intratumorally to half of the mice. The other half received PBS as a negative control. On day 23, mice were sacrificed.

### Statistical analysis and data visualization

Data were analyzed and plotted using GraphPad Prism (version 10.5.0). Specific information about data presentation is provided in each figure caption throughout the manuscript. Multiple illustrations were created with BioRender.

### Algorithm

Here we present the NeoADARgen—an algorithm that designs a patient-specific gRNA tailored to the individual’s unique tumor genetic characteristics. Owing to its precise design, using this gRNA as the main component of an endogenous-ADAR will induce A-to-I (G) edits at the tumor RNA level upon delivery to the tissue. The gRNA interacts exclusively with tumor cells, as it is designed to base-pair with a region containing somatic mutations that are absent in normal cells. Our algorithm identifies the optimal points for editing, which in turn alter the translated peptide within the cancer cell, creating a novel potent neoantigen that takes into account the individual’s HLA typing. We utilize a list of somatic mutations sequenced from the tumor and the patient’s HLA typing as basic inputs. For increased accuracy, incorporating tumor gene expression levels is also feasible.

#### Step 1: Ensuring binding to tumor cells while excluding normal cells

To generate neoantigens in tumor cells but not in normal cells, the initial step involves targeting a somatic mutation within the cancerous cell. This mutation can manifest in any possible variant type, as long as it modifies the RNA sequence in comparison to the wild type. The preliminary gRNA is programmed to bind by base pairing to a sequence comprising the mutation along with the 20 nucleotides upstream and the 20 nucleotides downstream, totaling in a sequence of ~40 nucleotides. Due to the presence of the somatic mutation at the core of the sequence, the gRNA binds to the cancerous cells but not to the normal cells upon approaching the tissue, as normal cells typically lack the tumor somatic variants. This step guarantees binding specificity exclusively to the tumor cells (Appendix Fig. S1).

#### Step 2: Identifying the optimal adenosines in the region for editing

The following step involves scanning the gRNA sequence for adenosines, which, upon conversion to guanosines, will modify the peptide in a manner predicted to generate a potent neoantigen. For this purpose, we evaluate each adenosine within the sequence to determine the effect of editing it to guanosine on the resulting protein. Given the natural ADAR’s ability to edit multiple adenosines in clusters, we also explore the impact of editing two adenosines at a time on the resulting protein. Note that adenosines preceded by guanosines are not considered, as they do not meet the criteria for the ADAR motif, which requires the absence of a guanosine 5’ to the edited adenosine. While this constraint can be overcome by manipulating the gRNA to include an additional mismatch 5’ to the edited adenosine, we prefer not to do so in order to maintain ideal complementarity to the cancerous sequence. Additionally, the examination excludes the first and last nucleotides of the sequence.

To identify the optimal alteration capable of generating a neoantigen, we analyzed all possible 9-mer subsequences derived from the gRNA sequence, where one or two adenosines are substituted with guanosines (Appendix Fig. S2). To assess the effect on the resultant peptide, we employed the external tool NetMHCpan4.1 (Reynisson et al, 2020), which utilizes artificial neural networks to predict peptide binding for a given sequence by a given HLA. We submitted each combination of altered subsequences along with each HLA class I type of the patient to the prediction tool, examining the output parameters for each combination. By default, thresholds of %Rank <0.5% and %Rank <2% are used to identify strong MHC binders (SBs) and weak MHC binders (WBs) for class I, respectively (Reynisson et al, 2020). In our analysis, we used even more conservative parameters: %Rank <0.1% and affinity <50 as indicators for SB. Additionally, if gene expression data were available, we set a threshold of TPM above 15 as an additional mandatory criterion for robustness. This ensures adequate transcription of the gene for RNA editors to function effectively. If more than one combination met these criteria, we prioritized the sub-sequence with the lowest detected rank.

#### Step 3: Presenting the best results

At the end, the algorithm presents all potential gRNA sequences, marking the optimal adenosines to edit with an asterisk (*). These gRNAs are the optimal sequences tailored for a given tumor sample, to be utilized with Endogenous-ADAR base editors. Upon delivery to the tissue, they will bind to a region encompassing a somatic mutation expressed in this tumor to ensure cancer cell specificity and induce single or double A-to-I editing, leading to precise peptide alterations. These alterations are predicted to generate neoantigens according to the patient’s HLA typing.

### Cancer databases

The computational results presented are based on cancer data extracted from the Tumor Cancer Genome Atlas (TCGA). To apply our algorithm to a variety of cancer samples, we utilized the Genomic Data Commons (GDC) data portal [https://portal.gdc.cancer.gov/]. We downloaded the data for seven cancer TCGA projects: BRCA, SKCM, OV, GBM, LIHC, LUAD, and LUSC. The first five were downloaded in September 2023, and the last two in January 2024. For each patient, we used the DNA sample as sequenced from the primary tumor or from the metastatic tumor if the primary sample was not available. In case the DNA file was not available, we used the whole genome sequence file (Appendix Table S2). For each patient, we also used the gene expression quantification file as sequenced from its tumor. Lastly, we used The Cancer Immunome Atlas (TCIA) data portal [https://tcia.at/home] to extract the HLA typing of each individual as reported by its unique TCGA ID. Only individuals with access to all data were included in our analysis.

To apply our algorithm to the most common cancer hotspots, we extracted a list of the most common somatic mutations and their reported prevalence across all cancer types as presented in GDC. We also downloaded all TCGA samples available in TCIA to aggregate the most common HLA types among them. Both lists were downloaded in September 2023. For this analysis, gene expression could not be considered by the algorithm, as the calculation was not done on an individual basis.

## Supplementary information

Appendix

Peer Review File

Source data Fig. 1

Source data Fig. 2

Source data Fig. 3

Source data Fig. 4

Source data Fig. 5

Expanded View Figures

## Data Availability

The source code used to produce the results and analyses presented in this manuscript is available on a GitHub repository at: https://github.com/landsboy/neoADARgen.git. The mass spectrometry data have been deposited with the ProteomeXchange Consortium (http://proteomecentral.proteomexchange.org; (Vizcaíno et al, 2014)) via the PRIDE partner repository, with dataset identifiers PXD077976 and JPST004598 for jPOS (Okuda et al, 2025). The source data of this paper are collected in the following database record: biostudies:S-SCDT-10_1038-S44318-026-00870-5.
