# Supplementary material for: Engineering editopes through programmable RNA editing toward tumor neoantigen generation
Source: EMBO J. 2026 Aug 14;45(18):6655–78. doi: 10.1038/s44318-026-00870-5 (PMC13578223; doi:10.1038/s44318-026-00870-5)
Supplement: Supplementary file 2 [file 44318_2026_870_MOESM2_ESM.pdf]

THE EMBO JOURNAL

Engineering editopes through programmable RNA editing toward tumor neoantigen generation

Riccardo Pecori, Beatrice Casati, Rona Merdler-Rabinowicz, Netanel Landesman, Khwab Sanghvi, Stefan Zens, Ece Bilgin, Mantiana Topouzi, Kai Kipfstuhl, Veronica Pinamonti, Annette Arnold, Mohammad Abukhalaf, David Gomez-Zepeda, Stefan Tenzer, John Lindner, Michael Platten, Rienk Offringa, Rafael Carretero, Eytan Ruppin, Erez Levanon, and F. Nina Papavasiliou

Corresponding author(s): F. Nina Papavasiliou (n.papavasiliou@dkfz.de) , Eytan Ruppin (Eytan.Ruppin@cshs.org), Erez Levanon (Erez.Levanon@biu.ac.il)

Review Timeline:

Submission Date:	24th Nov 25
Editorial Decision:	11th Feb 26
Revision Received:	11th May 26
Editorial Decision:	12th Jun 26
Revision Received:	21st Jun 26
Accepted:	22nd Jun 26

Editor: Ioannis Papaioannou

Transaction Report:

(Note: With the exception of the correction of typographical or spelling errors that could be a source of ambiguity, letters and reports are not edited. Depending on transfer agreements, referee reports obtained elsewhere may or may not be included in this compilation. Referee reports are anonymous unless the Referee chooses to sign their reports.)

Dear Nina,

Thank you again for submitting your manuscript EMBOJ-2025-123135 for consideration by The EMBO Journal, and for your patience during peer review. Your manuscript has been seen by three experts in the field, and we have now received the complete set of their reports, which are appended below. I have already shared with you the first two reports, and I would like to thank you for your quick feedback and for letting me know that you found the comments (of referees #1 and #2) reasonable and experimentally addressable.

As you will see, referee #3 also raises some significant concerns, some of which are shared with the other referees (for example, regarding the ability of the new system to generate novel immunogenic epitopes, which is a central claim in the manuscript), while we also find their point 3 (concerning the need for demonstration that the predicted neoepitopes can be stimulated and recognize natural tumor targets) particularly relevant. Other points of referee #3 would require clarification (point 1) or more detailed discussion (points 4 and 5).

On balance, and taking into consideration the supportive input we received from the referees with regard to the overall significance of the topic and the novelty of the approach, as well as the fact that you informed us that you are already addressing some of the major concerns experimentally, I would like to invite you to submit a thoroughly revised version of your manuscript taking the referees' suggestions on board, along with a detailed point-by-point response addressing all referees' comments. Please note that it is The EMBO Journal policy to allow only a single round of major revision, and acceptance of your manuscript will therefore depend on the completeness of your responses in this revised version. Given the nature of the raised concerns, I think that it would be useful to discuss your revision plan already during the initial phase of your revision. You are very welcome to share with me a draft point-by-point response/revision plan explaining if there are any points you do not agree with or cannot address, and we could then arrange a video call to discuss further, if necessary.

We generally allow three months as standard revision time (10 May, 2026). As a matter of policy, competing manuscripts published during this period will not negatively impact our assessment of the conceptual advance presented by your study. However, we request that you contact us as soon as possible upon publication of any related work, to discuss how to proceed. Should you foresee a problem in meeting this three-month deadline, please let us know in advance and we will be able to grant an extension.

Please let me know if you have any questions or comments that you would like to discuss with me. Thank you for the opportunity to consider your work for publication in The EMBO Journal. I look forward to your revision.

Best regards,

Ioannis

Ioannis Papaioannou, PhD
Editor, The EMBO Journal
i.papaioannou@embojournal.org

*****
Instructions for preparing your revised manuscript
*****

1. When you are ready to submit the revision, please upload:

- A Word file of the manuscript text (including legends of main Figures, EV Figures and Tables). Please make sure that changes are highlighted (or "tracked") to be clearly visible.

- Individual production-quality figure files (one file per figure). When assembling your figures, please refer to our figure preparation guidelines in order to ensure proper formatting and readability in print as well as on screen:
https://bit.ly/EMBOPressFigurePreparationGuideline
If the data shown in a figure are obtained from n {less than or equal to} 2, please use scatter plots showing the individual data points.
Please also include scale bars in all microscopy images.
The following points must be specified in each figure legend:
i. the name of the statistical test used to generate error bars and P values
ii. the number (n) of independent experiments (please specify technical or biological replicates) underlying each data point

(discussion of statistical methodology can be reported in the Materials and Methods section, but figure legends should contain a basic description of n, P, and the test applied)
iii. the nature of the bars and error bars (s.d., s.e.m.).
See also guidelines for figure legends: https://www.embopress.org/page/journal/14602075/authorguide#figureformat

- A point-by-point response to the referees' comments, with a detailed description of the changes made (as a word file). All referees' concerns must be fully addressed and their suggestions taken on board. When preparing your letter of response to the referees' comments, please bear in mind that this will form part of the Review Process File and will therefore be available online to the community. Please note that you have the possibility to opt out of the transparent process at any stage prior to publication by letting the editorial office know (contact@embojournal.org); if you do opt out, the Review Process File link will point to the following statement: "No Review Process File is available with this article, as the authors have chosen not to make the review process public in this case.". For more details on our Transparent Editorial Process, please visit our website: https://www.embopress.org/page/journal/14602075/authorguide#transparentprocess

- Expanded View (EV) files (replacing Supplementary Information) that are collapsible/expandable online. A maximum of 5 EV Figures can be typeset. EV Figures should be cited as "Figure EV1, Figure EV2" etc. in the text, and their respective legends should be included in the manuscript file after the legends of regular figures. See detailed instructions regarding Expanded View files here:
https://www.embopress.org/page/journal/14602075/authorguide#expandedview

- For the figures that you do NOT wish to display as Expanded View figures, they should be bundled together with their legends in a single PDF file called "Appendix", which should start with a short Table of Contents (including page numbers). Appendix figures should be referred to in the main text as: "Appendix Figure S1, Appendix Figure S2" etc. Please see detailed instructions here: https://www.embopress.org/page/journal/14602075/authorguide#expandedview

- A complete author checklist, which you can download from our author guidelines (https://www.embopress.org/page/journal/14602075/authorguide). Please note that the checklist will also be part of the Review Process File.

2. Please note that no statistics should be calculated and shown in Figures if n=2. Please also note that each p value should be reported as an exact value.

3. Before submitting your revision, primary datasets (and computer code, where appropriate) produced in this study need to be deposited in appropriate public databases (see https://www.embopress.org/page/journal/14602075/authorguide#dataavailability). In particular, all datasets such as DNA or RNA sequencing data, or mass spectrometry data generated in the study must be deposited in appropriate repositories. The accession numbers, database, and the specific URLs (links) should be listed in a formal "Data availability" section (placed after Methods), following the example below:

"The RNA-seq datasets produced in this study are available in the following database:
Gene Expression Omnibus GSE46843 (https://www.ncbi.nlm.nih.gov/geo/query/acc.cgi?acc=GSE46843)"

*** All links should resolve to a page where the data can be accessed. ***

*** Please remember to provide in the Data availability section of your revised manuscript reviewer passwords if the datasets are not yet public. ***

*** The Data Availability Section is restricted to new primary data that are part of this study. In case you have no data that require deposition in a public database, please state so instead of referring to the database: "Our study includes no data deposited in public repositories." under the heading "Data availability". ***

4. The materials and methods need to be described in the manuscript using our structured methods format, which is now required for all research articles. According to this format, the Methods section includes a single "Reagents and Tools Table" - listing key reagents, experimental models, software and relevant equipment including their sources and relevant identifiers- followed by a "Methods and Protocols" section describing the methods. Please download and fill our Reagents and Tools Table template (.docx), which you can find in our author guide:
https://www.embopress.org/page/journal/14602075/authorguide#structuredmethods. When submitting your revised manuscript, please do not include the Reagents and Tools Table in the Methods section of the manuscript but instead upload it as a separate file choosing the file type "Reagent Table".

5. Please check that the title and the abstract of the manuscript are brief, yet explicit, even to non-specialists. The length of the title should not exceed 100 characters, and the abstract should be a single paragraph not exceeding 175 words.

6. Please also note our reference format: https://www.embopress.org/page/journal/14602075/authorguide#referencesformat.

7. At EMBO Press we ask authors to provide source data for the main manuscript figures. You will receive a separate email with instructions for providing source data with your revised manuscript, including how to upload and organize the files.

8. Please remember: digital image enhancement is acceptable practice, as long as it accurately represents the original data and conforms to community standards. If a figure has been subjected to significant electronic manipulation, this must be noted in the figure legend or in the "Materials and Methods" section. The editors reserve the right to request original versions of figures and the original images that were used to assemble the figure.

9. Our journal encourages inclusion of data citations in the reference list to directly cite datasets that were obtained from public databases. Data citations in the article text are distinct from normal bibliographical citations and should directly link to the database records from which the data can be accessed. In the main text, data citations are formatted as follows: "Data ref: Smith et al, 2001" or "Data ref: NCBI Sequence Read Archive PRJNA342805, 2017". In the Reference list, data citations must be labeled with "[DATASET]". A data reference must provide the database name, accession number/identifiers, and a resolvable link to the landing page from which the data can be accessed at the end of the reference. Further instructions are available at: https://www.embopress.org/page/journal/14602075/authorguide#referencesformat.

10. We request authors to consider both actual and perceived competing interests. Please review our policy (https://www.embopress.org/page/journal/14602075/authorguide#conflictsofinterest) and update your competing interests statement if necessary. Please name this section 'Disclosure and competing interests statement' and place it after the Acknowledgements section.

11. Please note that all corresponding authors are required to provide an ORCID ID upon submission of a revised manuscript (https://orcid.org/). Please find instructions on how to link your ORCID ID to your account in our manuscript tracking system in our Author guidelines
(https://www.embopress.org/page/journal/14602075/authorguide#authorshipguidelines).

12. We use CRediT to specify the contributions of each author in the journal submission system. CRediT replaces the author contribution section, which should be removed from the manuscript. Please use the free text box to provide more detailed descriptions. See also guide to authors:
https://www.embopress.org/page/journal/14602075/authorguide#authorshipguidelines.

13. Further information is available in our Guide For Authors: https://www.embopress.org/page/journal/14602075/authorguide

14. We would also welcome the submission of cover suggestions or motifs to be used by our Graphics Illustrator in designing a cover.

15. Please use the link below to submit your revision:
https://emboj.msubmit.net/cgi-bin/main.plex

Referee #1:

Here the authors describe the novel concept of using A to I RNA editing to create amino acid changes in peptide sequences to create new antigenic peptides for tumor targeting in immunotherapy. Their approach has two important features. First, guide RNAs are designed that can recruit ADAR enzymes to make changes in codons and thereby generate novel peptides for display on the tumor surface. Secondly, they describe a computational platform to predict guide RNAs specific for A to I sites near somatic mutations that would only exist in the tumor cells and not normal cells of the patient. The overall approach is very compelling, and their initial results are encouraging. For instance, they have clearly shown that they are able to use ADAR guide RNAs to restore a known immunogenic peptide sequence (MART-1) from a non-immunogenic variant and that only a modest level of editing (~20%) can lead to T-cell activation in their model in vitro system. They also show T-cell targeting of tumors in mice that are derived from mutant MART-1 expressing cells also expressing the specific RNA editing guide strand needed to regenerate the functional epitope. The paper is clear and well written. The topic is important. However, the authors have not provided experimental evidence to support their claims to have used RNA editing to generate tumor-specific and new immunogenic peptide sequences as their title states. The manuscript requires additional experimental validation before this claim can be made. I have the following specific comments.

1) At this point, the authors have not demonstrated that A to I editing can create new immunogenic peptide sequences, only restore a previously known epitope sequence (MART-1). This is related to their claim to have generated neoantigens.

2) They have not demonstrated specificity for editing only in transcripts containing tumor-specific somatic mutations. This is related to their claim of tumor-specificity.
3) It also should be noted that these authors have engineered cells to express both the MART-1 peptide and either a non-targeting or targeting guide RNA for directed RNA editing. In their in vivo tests, the mice receive cells that express either WT MART-1 or mutant MART-1 from which the tumors are derived. The mutant MART-1 expressing cells are also engineered to express the editing guide strand. The mice with tumors then received adoptive T-cell therapy with MART-1 specific T cells. This system is substantially different from a therapeutic scenario where a guide RNA must be efficiently delivered to a pre-existing tumor.

Referee #2:

Review of Pecori et al.,

In this manuscript, the authors aim at introducing neo-antigens into tumor cells to elicit T-cell recognition. They aim to do so by recruiting endogenous ADAR1 to target a model antigen in melanomas (MART) that was previously mutated to become immunologically silent.

To optimize guide RNAs a reporter assay that builds on correcting a mutation in GFP is used to identify optimal guide RNAs. In doing so, rules for the efficient construction of guides are established. Using this technology, 74nt long guides are established. Next, using the rules identified to design efficient guides, a guide RNA against MART is developed that allows the change of a G to a S codon.
A MART gene, expressed in the immunologically silent G versio is initially expressed in Hek cells. Upon editing to 20% (S codon) the cells are efficiently targeted by T cells expressing the corresponding DMF5 TCR.

Next, the system is transferred to a melanoma cell model where 10% editing is observed upon guide RNA expression. Upon injection into mice, tumors developed that were efficiently targetd by T cells expressing the appropriate T cell receptor.

Finally, a bioinformatic approach is chosen to identify putative neoantigens that could be introduced by A to I editing. Upon screening of the TCGA dataset, an average of 4 to 6 neoantigens per tumor could theoretically be introduced. Most interestingly, such neoeptopees are sometimes shared between patients, such as a conserved site in KRAS.

Overall, the manuscript is very well and clear written and the data supports the data. Clearly, the efficiency of the system may be a bottleneck when it comes to clinical application and this is also discussed. However, the authors should be more clear about this.

• Specifically, the expression of the guide RNA should be better documented. The materials and methods state that a piggy bac system was employed for stable integration. This should also be mentioned in the text. Also, levels of guide RNA expression should be quantified. As a U6 promoter is used, relatively strong expression will be achieved.
• The U6 promoter will generate nuclear RNAs (unless fused to export sequences). Can the authors comment on the site of editing? While the HEK293 cells used for the guide uptimization are claimed to only express ADAR1, it is not clear whether the melanome cells used, also only express ADAR1. Please comment.
• Expression of MART, how strong is the expression of MART in the melanoma cells. Is this a highly expressed antigen? Please comment.

Minor: in the Materials and Methods, a section on optimized APOBECs is included, which seemingly does not belong to this version of the manuscript. This should be removed.

Referee #3:

This manuscript presents an approach for generating de novo mutations in tumor cells that can then potentially elicit tumor-reactive T cells. There were several issues, however, that need to be further addressed in this study.
1. It appeared that in order to specifically edit tumor cells and not normal cells that there needed to be a mutation within 75 bp of the region that was selected for alteration. It was not clear why the 4 nucleotide mis-match 35 nucleotides downstream of the target site was allowed and actually facilitated editing. If that is true, why does the inclusion of a mutant site within the construct help to insure the specific editing of tumor cells?
2. These results demonstrate the ability of this system to re-generate a known epitope that has been edited in a way that renders the sequence non-immunogenic but there was no evidence that a novel immunogenic neoepitope could be generated using this system. There was a discussion of this approach at the end of the Results section but this appears to have been an in silico demonstration that a potentially immunogenic epitope could be engineered into a tumor.
3. While the HLA binding prediction tools allow one to identify novel neoepitope candidates, the majority of these epitopes are

not likely to be naturally processed and presented. Thus, a large expanded study would be required to demonstrate that the predicted neoepitopes could be stimulated and would also recognize natural tumor targets.
4. The relatively low editing ability of this system would mean that in vivo only a small proportion of tumor cells could potentially be modified to express the mutant editope. Unmodified tumor cells would not therefore be susceptible to recognition and lysis and thus it is unlikely that this could result in an effective anti-cancer treatment.
5. Additional ways that these methods could be applied to the development of cancer therapies should be included in the Discussion section.

Response to referees' letter

DEUTSCHES KREBSFORSCHUNGSZENTRUM IN DER HELMHOLTZ-GEMEINSCHAFT

May 11th, 2026

Dear Reviewers,

Thank you for reviewing our manuscript entitled "**Engineering *editopes* through programmable RNA editing toward tumor neoantigen generation"** for publication in *The EMBO Journal*. We are grateful for the insightful comments and critiques, which helped us improve the manuscript substantially. We have addressed all comments point by point below.

All changes related to the comments are highlighted in yellow in the revised manuscript.

We look forward to your decision.

With best regards,

Dr. Riccardo Pecori & Prof. Nina Papavasiliou
(for the authors)

Response to Reviewers' comments

Referee #1:

Here the authors describe the novel concept of using A to I RNA editing to create amino acid changes in peptide sequences to create new antigenic peptides for tumor targeting in immunotherapy. Their approach has two important features. First, guide RNAs are designed that can recruit ADAR enzymes to make changes in codons and thereby generate novel peptides for display on the tumor surface. Secondly, they describe a computational platform to predict guide RNAs specific for A to I sites near somatic mutations that would only exist in the tumor cells and not normal cells of the patient. The overall approach is very compelling, and their initial results are encouraging. For instance, they have clearly shown that they are able to use ADAR guide RNAs to restore a known immunogenic peptide sequence (MART-1) from a non-immunogenic variant and that only a modest level of editing (~20%) can lead to T-cell activation in their model in vitro system. They also show T-cell targeting of tumors in mice that are derived from mutant MART-1 expressing cells also expressing the specific RNA editing guide strand needed to regenerate the functional epitope. The paper is clear and well written. The topic is important. However, the authors have not provided experimental evidence to support their claims to have used RNA editing to generate tumor-specific and new immunogenic peptide sequences as their title states. The manuscript requires additional experimental validation before this claim can be made. I have the following specific comments.

Response: We thank the reviewer for the thoughtful summary and for highlighting the need for experimental validation of neoepitope generation. We agree that our original data were limited to the restoration of a known epitope (MART-1) and did not directly demonstrate the generation of novel immunogenic peptide sequences.

To address this point, we have now included new experimental data demonstrating that SPEAR gRNAs can generate known immunogenic neoepitopes at the RNA level. In addition, we have revised the title and text throughout the manuscript to more accurately reflect the scope of our experimental evidence.

1) At this point, the authors have not demonstrated that A to I editing can create new immunogenic peptide sequences, only restore a previously known epitope sequence (MART-1). This is related to their claim to have generated neoantigens.

Response: We thank the reviewer for highlighting this discrepancy between the data and claims. We have now included new data demonstrating that SPEAR gRNAs can generate known immunogenic neoantigens at the RNA level. Specifically, we targeted endogenous sites corresponding to previously described immunogenic neoepitopes (including TP53^{Y220C},

USP9X^{Y2009C}, and C6orf89^{Y813C}) and showed that precise A-to-I editing can recapitulate the corresponding amino acid substitutions in both HEK293T and 624-mel cells. These results demonstrate that SPEAR-mediated RNA editing is not limited to restoring disrupted epitopes but can also be used to generate immunogenic neoepitopes beyond the MART-1 model system (new Figure 2 and related text).

2) They have not demonstrated specificity for editing only in transcripts containing tumor-specific somatic mutations. This is related to their claim of tumor-specificity.

Response: We thank the reviewer for raising this important point. Our platform relies on sequence-specific hybridization, a principle widely used in many established molecular technologies that can discriminate even single-nucleotide differences. For example, single-nucleotide specificity is routinely achieved in technologies such as microarrays, allele-specific PCR, CRISPR guide RNA targeting, molecular beacons, and SNP genotyping assays. These approaches demonstrate that hybridization-based strategies can be designed to distinguish sequences differing by only a single nucleotide. Importantly, while our framework is designed to target tumor-specific somatic mutations, the degree of specificity can be further optimized through careful guide design and hybridization parameters. In cases where the somatic variant involves larger sequence differences - such as dinucleotide or trinucleotide substitutions, insertions, or deletions - the theoretical specificity of hybridization-based targeting is expected to be even higher. We agree that experimental validation will ultimately be required to quantify the specificity of editing in cellular systems, and we have clarified this point in the revised manuscript (see lines 396-399).

3) It also should be noted that these authors have engineered cells to express both the MART-1 peptide and either a non-targeting or targeting guide RNA for directed RNA editing. In their in vivo tests, the mice receive cells that express either WT MART-1 or mutant MART-1 from which the tumors are derived. The mutant MART-1 expressing cells are also engineered to express the editing guide strand. The mice with tumors then received adoptive T-cell therapy with MART-1 specific T cells. This system is substantially different from a therapeutic scenario where a guide RNA must be efficiently delivered to a pre-existing tumor.

Response: We thank the reviewer for pointing out this limitation in our study. We agree with the reviewer that the experimental setup used in this study does not fully recapitulate a therapeutic scenario in which guide RNAs would need to be delivered to an established tumor *in vivo*. This represents an important limitation of the current work. The primary aim of our study was to establish and rigorously evaluate the *conceptual feasibility* of using targeted RNA editing to generate immunogenic epitopes in tumor cells, independently of the additional technical constraints imposed by *in vivo* delivery. To this end, we deliberately employed a controlled system in which guide RNA

expression was ensured, allowing us to isolate and assess the immunological consequences of SPEAR-mediated RNA editing per se. We fully agree that efficient and tumor-selective delivery of guide RNAs will be a critical requirement for clinical translation. Addressing this challenge is an essential next step and is currently the focus of ongoing work in our laboratory, which we plan to report separately.

To explicitly acknowledge this limitation, we have revised the Discussion section to clarify that the current *in vivo* experiments are intended as a proof-of-principle demonstration and to highlight delivery as a key outstanding challenge for future therapeutic applications (see lines 428-435).

Referee #2:

In this manuscript, the authors aim at introducing neo-antigens into tumor cells to elicit T-cell recognition. They aim to do so by recruiting endogenous ADAR1 to target a model antigen in melanomas (MART) that was previously mutated to become immunologically silent.

To optimize guide RNAs a reporter assay that builds on correcting a mutation in GFP is used to identify optimal guide RNAs. In doing so, rules for the efficient construction of guides are established. Using this technology, 74nt long guides are established. Next, using the rules identified to design efficient guides, a guide RNA against MART is developed that allows the change of a G to a S codon.

A MART gene, expressed in the immunologically silent G versio is initially expressed in Hek cells. Upon editing to 20% (S codon) the cells are efficiently targeted by T cells expressing the corresponding DMF5 TCR.

Next, the system is transferred to a melanoma cell model where 10% editing is observed upon guide RNA expression. Upon injection into mice, tumors developed that were efficiently targetd by T cells expressing the appropriate T cell receptor.

Finally, a bioinformatic approach is chosen to identify putative neoantigens that could be introduced by A to I editing. Upon screening of the TCGA dataset, an average of 4 to 6 neoantigens per tumor could theoretically be introduced. Most interestingly, such neoeptopees are sometimes shared between patients, such as a conserved site in KRAS.

Overall, the manuscript is very well and clear written and the data supports the data. Clearly, the efficiency of the system may be a bottleneck when it comes to clinical application and this is also discussed.

Response: We thank the reviewer for the careful evaluation of our manuscript and for the constructive summary. We have addressed each of the specific comments and suggestions in detail below.

However, the authors should be more clear about this.

• Specifically, the expression of the guide RNA should be better documented. The materials and methods state that a piggy bac system was employed for stable integration. This should also be

mentioned in the text. Also, levels of guide RNA expression should be quantified. As a U6 promoter is used, relatively strong expression will be achieved.

Response: We thank the reviewer for this suggestion. We have revised the main text to explicitly clarify that stable expression of the guide RNA was achieved using a Sleeping Beauty transposon-based system (see lines 259-260 in the revised manuscript), as already described in the Materials and Methods.

In addition, we have now quantified guide RNA expression levels by RT-qPCR. These data confirm robust guide RNA expression driven by the U6 promoter and have been included in the revised manuscript (updated Figure S3, new panel E). Due to technical limitations in primer design, we were able to reliably detect only the NT-gRNA; however, both NT- and T-gRNAs are expressed from the same transposon backbone (Figure S3B) and show comparable integration rates, as assessed by eBFP expression (Figure S3D), supporting similar expression levels between NT- and T-gRNAs.

Furthermore, RT-qPCR analysis allowed us to compare gRNA expression with that of the target MLANA transcript. This analysis indicates that the gRNA is expressed at lower levels than MLANA (~60-fold difference), providing additional context for the observed editing efficiencies (see new Figure S3E and accompanying text).

Together, these data improve the characterization and transparency of guide RNA expression in our experimental system. We have updated the manuscript accordingly (see lines 262-269 in the revised manuscript) and provided technical details on the method and results for RT-qPCR of gRNAs in the supplementary information file (see Supplementary Methods section).

• The U6 promoter will generate nuclear RNAs (unless fused to export sequences). Can the authors comment on the site of editing? While the HEK293 cells used for the guide uptimization are claimed to only express ADAR1, it is not clear whether the melanome cells used, also only express ADAR1. Please comment.

Response: We thank the reviewer for raising this important point regarding guide RNA localization and ADAR enzyme expression. Consistent with the use of a U6 promoter, guide RNAs are expected to localize predominantly to the nucleus, as suggested by the reviewer and confirmed by published literature, see Extended Figure 1d from (Katrekar et al., 2022) (https://www.nature.com/articles/s41587-021-01171-4) and Supplementary Figure S9 in (Reautschnig et al., 2022) (https://www.nature.com/articles/s41587-021-01105-0). Accordingly, RNA editing mediated by SPEAR gRNAs is anticipated to occur primarily in the nuclear compartment, independently of which ADAR is expressed.

To directly address the identity of the editing enzyme in the cell lines used in our study, we have now experimentally confirmed that RNA editing in our HEK293T reporter system is strictly

ADAR1-dependent. We repeated the reporter assay experiment using ADAR1 wild-type and ADAR1 knockout HEK293T cells stably expressing the reporter. The KO cell line was previously generated (**see clone 3 in Supplementary Figure 7 from (Pecori et al., 2023).**

In this experiment, we transfected the optimized SPEAR gRNA targeting eGFP^{W58X}, encoded in the pT vector (used *in vivo* as the NT-gRNA control; see Figure S3 for the plasmid schematic), enabling detection of transfected cells via eBFP expression. Editing activity was observed exclusively in ADAR1 wild-type cells and was completely abolished in ADAR1 knockout cells, despite effective plasmid transfection in both cell lines, as measured by eBFP (see new panel F in the updated version of Figure S1). Additionally, to avoid bias due to differences in dual-reporter expression, we employed an ADAR1-knockout HEK293T clone with a comparable mCherry expression level to that of the ADAR1 wt clone (see new panel G in the updated version of Figure S1). Taken together, the results of this experiment strongly support the conclusion that ADAR2 does not contribute to editing in this reporter system, making our gRNA optimization ADAR1 specific. We have now updated the manuscript text to express this concept (lines 146-147 and 172-182).

Finally, to address the reviewer's question regarding the melanoma cell line, we have now assessed ADAR1 and ADAR2 expression in 624-mel cells by RT–qPCR. ADAR1 expression is approximately 17-fold higher than ADAR2 in 624-mel cells, indicating that ADAR1 is the predominant ADAR family member expressed in this cell line, supporting the conclusion that SPEAR-mediated editing in melanoma cells is mostly ADAR1-driven (see updated Figure S3, new panel E). We have updated the manuscript text to express this concept (lines 269-271).

• Expression of MART, how strong is the expression of MART in the melanoma cells. Is this a highly expressed antigen? Please comment.

Response: We thank the reviewer for this question. MART-1 (MLANA) is a highly expressed melanoma-associated antigen. Publicly available transcriptomic datasets indicate high MLANA expression in melanoma, with levels reaching approximately 850 TPMs in 624-mel, as documented in the Expression Atlas.

To directly confirm expression in our experimental system, we quantified MLANA expression in 624-mel cells expressing either targeting (T) or non-targeting (NT) SPEAR gRNAs by RT-qPCR. This analysis confirmed robust MART-1 expression in these melanoma cell lines (approximately 2.5-fold higher than PGK1, see updated Figure S3, new panel E), consistent with its well-established role as a melanoma differentiation antigen. Importantly, the expression of targeting gRNA did not affect MART-1 expression level, indicating that SPEAR gRNA binding does not induce transcript silencing. We have updated the manuscript accordingly (lines 261-269).

Minor: in the Materials and Methods, a section on optimized APOBECs is included, which seemingly does not belong to this version of the manuscript. This should be removed.

Response: We thank the reviewer for pointing this out; it was not a mistake. Here we'd like to explain it better since it was unclear. In our base editing experiments aimed at introducing the genomic mutation in the gene encoding MART-1, as described in our manuscript, we tested different base editors before achieving the desired result, starting with the wild-type cytosine base editor APOBEC1. However, as we observed off-target editing events, we optimized the experiment by testing different modified versions of the enzyme already described in the literature. In particular, Doman and colleagues (Doman et al., 2020, ref #52 in the manuscript) engineered a version of APOBEC1 that minimizes off-target DNA editing, which proved efficient and precise in our experiments as well. For clarity, we highlighted this detail in our Materials and Methods (lines 678-680 of the submitted version) to ensure our experiments can be promptly reproduced.

Referee #3:

This manuscript presents an approach for generating de novo mutations in tumor cells that can then potentially elicit tumor-reactive T cells. There were several issues, however, that need to be further addressed in this study.

1. It appeared that in order to specifically edit tumor cells and not normal cells that there needed to be a mutation within 75 bp of the region that was selected for alteration. It was not clear why the 4 nucleotide mis-match 35 nucleotides downstream of the target site was allowed and actually facilitated editing. If that is true, why does the inclusion of a mutant site within the construct help to insure the specific editing of tumor cells?

Response: We thank the reviewer for this important question and appreciate the opportunity to clarify this point. The 4-nucleotide mismatch centered ~35 nucleotides downstream of the editing site and the tumor-specific mutation serve fundamentally different purposes. The downstream mismatch is an engineered structural feature that improves ADAR1 recruitment and positioning, as previously described for LEAPER gRNA systems ((Uzonyi et al., 2021) https://pubmed.ncbi.nlm.nih.gov/33905683/). Importantly, this mismatch is located outside the critical pairing region surrounding the edited adenosine and therefore does not compromise overall guide RNA binding.

In contrast, tumor specificity relies on sequence complementarity between the guide RNA and the target transcript at the region containing the tumor mutation. The presence of the mutation allows stable duplex formation, whereas the corresponding wild-type transcript contains mismatches at this site that reduce hybridization stability and strongly impair editing efficiency. Thus, the tumor-associated mutation acts at the level of target recognition, while the downstream engineered mismatch modulates editing efficiency after guide RNA binding has occurred.

We have revised the manuscript to clarify this distinction and to better explain how structural mismatches that enhance ADAR activity can coexist with sequence-dependent tumor specificity (see lines 392-399).

2. These results demonstrate the ability of this system to re-generate a known epitope that has been edited in a way that renders the sequence non-immunogenic but there was no evidence that a novel immunogenic neoepitope could be generated using this system. There was a discussion of this approach at the end of the Results section but this appears to have been an in silico demonstration that a potentially immunogenic epitope could be engineered into a tumor.

Response: We thank the reviewer for highlighting this discrepancy between the data and claims. We have now included new data demonstrating that SPEAR gRNAs can generate known immunogenic neoantigens at the RNA level. Specifically, we targeted endogenous sites

corresponding to previously described immunogenic neoepitopes (including TP53^{Y220C}, USP9X^{Y2009C}, and C6orf89^{Y813C}) and showed that precise A-to-I editing can recapitulate the corresponding amino acid substitutions in both HEK293T and 624-mel cells. These results demonstrate that SPEAR-mediated RNA editing is not limited to restoring disrupted epitopes but can also be used to generate immunogenic neoepitopes beyond the MART-1 model system (new Figure 2 and related text).

3. While the HLA binding prediction tools allow one to identify novel neoepitope candidates, the majority of these epitopes are not likely to be naturally processed and presented. Thus, a large expanded study would be required to demonstrate that the predicted neoepitopes could be stimulated and would also recognize natural tumor targets.

Response: We thank the reviewer for highlighting this important point. We agree that HLA-binding prediction tools primarily identify candidate neoepitopes, and that many predicted peptides may ultimately not be naturally processed, presented, or recruit T cells (ie, be immunogenic) *in vivo*. This section of the study is intended as a bioinformatic framework for identifying potential neoantigen candidates rather than demonstrating functional immunogenicity. As the reviewer correctly notes, neoantigen prediction remains challenging and in silico methods are inherently imperfect; therefore, predicted epitopes require experimental validation to confirm natural processing, presentation, and T cell recognition. We have now added this limitation to the Discussion section and clarified that the neoepitopes identified in our analysis represent candidates that would require further experimental validation in future studies (see lines 400-413 in the updated manuscript).

4. The relatively low editing ability of this system would mean that in vivo only a small proportion of tumor cells could potentially be modified to express the mutant editope. Unmodified tumor cells would not therefore be susceptible to recognition and lysis and thus it is unlikely that this could result in an effective anti-cancer treatment.

Response: We thank the reviewer for raising this important point. We apologize for not having explained this aspect more clearly in the original manuscript. There are two aspects to this and we will clarify them separately:

1) Heterogeneity of editing between cells. Our current data suggest that the observed ~10% editing does not reflect the presence of a small subpopulation of highly edited cells within an otherwise unedited cell population. Rather, the data are consistent with an average editing level of approximately 10% per cell across the population. All melanoma cells used in these experiments were BFP-positive and selected for

blasticidin resistance (see Fig. S3D), indicating stable integration and uniform expression of the guide RNA cassette (see Fig. S3B).

In addition, both our *in vitro* (Fig. 4C in the revised manuscript) and *in vivo* (Fig. 4E and Fig. S3G in the revised manuscript) results argue against the presence of a large fraction of completely unedited cells. If the measured 10% editing represented only a small edited subpopulation (e.g., ~10% edited cells and ~90% unedited cells), the latter would be expected to escape T cell recognition and continue proliferating, resulting in detectable tumor outgrowth. This was not observed.

Therefore, our results indicate that relatively modest levels of RNA editing (~10%) within cancer cells are sufficient to trigger effective T cell responses when SPEAR-gRNAs are expressed in tumor cells.

2) <u>Heterogeneity in the delivery of ADAR-recruiting guide RNAs</u>. We agree that efficient delivery of guide RNAs to tumor cells *in vivo* remains a key challenge for therapeutic translation. This issue is distinct from editing efficiency per cell and will be addressed separately in future work focused on delivery strategies.

5. Additional ways that these methods could be applied to the development of cancer therapies should be included in the Discussion section.

Response: We thank the reviewer for bringing this up. We have now included a new paragraph in the discussion section describing how this approach could be coupled with other cancer therapies (see lines 436-442)

References

Doman, J.L., Raguram, A., Newby, G.A., Liu, D.R., 2020. Evaluation and minimization of Cas9-independent off-target DNA editing by cytosine base editors. Nat Biotechnol 38, 620–628. https://doi.org/10.1038/s41587-020-0414-6

Katrekar, D., Yen, J., Xiang, Y., Saha, A., Meluzzi, D., Savva, Y., Mali, P., 2022. Efficient in vitro and in vivo RNA editing via recruitment of endogenous ADARs using circular guide RNAs. Nat Biotechnol 40, 938–945. https://doi.org/10.1038/s41587-021-01171-4

Pecori, R., Chillón, I., Lo Giudice, C., Arnold, A., Wüst, S., Binder, M., Marcia, M., Picardi, E., Papavasiliou, F.N., 2023. ADAR RNA editing on antisense RNAs results in apparent U-to-C base changes on overlapping sense transcripts. Frontiers in Cell and Developmental Biology 10.

Reautschnig, P., Wahn, N., Wettengel, J., Schulz, A.E., Latifi, N., Vogel, P., Kang, T.-W., Pfeiffer, L.S., Zarges, C., Naumann, U., Zender, L., Li, J.B., Stafforst, T., 2022. CLUSTER guide RNAs enable precise and efficient RNA editing with endogenous ADAR enzymes in vivo. Nat Biotechnol 40, 759–768. https://doi.org/10.1038/s41587-021-01105-0

Uzonyi, A., Nir, R., Shliefer, O., Stern-Ginossar, N., Antebi, Y., Stelzer, Y., Levanon, E.Y., Schwartz, S., 2021. Deciphering the principles of the RNA editing code via large-scale systematic probing. Mol Cell 81, 2374-2387.e3. https://doi.org/10.1016/j.molcel.2021.03.024

1st Revision - Editorial Decision 12th Jun 2026

Dear Nina,

Thank you again for the submission of your revised manuscript (EMBOJ-2025-123135R) to The EMBO Journal for our consideration, and for your patience during re-review. Your manuscript and your point-by-point responses to the comments of all original referees have now been seen by referees #2 and #3, and we have received their comments, which are appended below.

As you will see, both referees are satisified with the revision and find all initially raised points and criticisms adequately addressed. In light of this input and their recommendations, I am pleased to inform you that your manuscript has been accepted in principle for publication in The EMBO Journal.

Before we can proceed with the next steps towards publication of your article, there is a minor comment regarding an incorrect citation (referee #2), which we kindly ask you to address in a final version of your manuscript and in a brief response to the referee's suggestion.

In addition, there are a few more changes and corrections from the editorial side we need you to make in the final version of the manuscript:

- We noticed that four co-authors are marked as co-corresponding authors of the revised version of the manuscript, which is unusual for our journal. We kindly request that you review carefully again the contributions of each co-author and consider limiting the corresponding author status to no more than three co-authors. Please also note that we use the CRediT author contributions taxonomy system for specifying the contributions of each co-author, and detailed descriptions can also be provided if deemed useful (please see also below for more information).

- Please make sure that an institutional e-mail address is provided for each one of the co-corresponding authors of the final version of the manuscript below the authors' affiliations section (title page).

- Our notifications to the e-mail address r.carretero@Dkfz-Heidelberg.de of your co-author Rafael Carretero could not be delivered. Please make sure that your co-author updates their profile in our system with a valid e-mail address or send us the current/new e-mail address so that we can update the account on your co-author's behalf.

- All funding information provided in the Acknowledgements section of the manuscript should also be entered in our online manuscript handling system; information about the "Helmholtz Association" is currently missing in the online system.

- The reference format must be updated to the style used by EMBO Press (i.e., alphabetical list, with the names of the first 10 co-authors listed for each citation, followed by "et al."). For more information, please refer to our Author Guidelines: https://link.springer.com/journal/44318/submission-guidelines#cms-Reference-guidelines.

- Before submitting your revision, primary datasets (and computer code, where appropriate) produced in this study need to be deposited in appropriate public databases (see https://link.springer.com/partners/embo-press/editorial-policies#Data%20availability%20statement). In particular, all datasets such as DNA or RNA sequencing data, or mass spectrometry data generated in the study must be deposited in appropriate repositories. The accession numbers, database, and the specific URLs (links) should be listed in a formal "Data availability" section (placed after Methods), following the example below:

"The RNA-seq datasets produced in this study are available in the following database:
Gene Expression Omnibus GSE46843 (https://www.ncbi.nlm.nih.gov/geo/query/acc.cgi?acc=GSE46843)"

*** All links should resolve to a page where the data can be accessed. ***
*** Please remember to provide in the Data availability section of your revised manuscript reviewer passwords if the datasets are not yet public. ***
*** The Data Availability Section is restricted to new primary data that are part of this study. In case you have no data that require deposition in a public database, please state so instead of referring to the database: "Our study includes no data deposited in public repositories." under the heading "Data availability". ***

- Please change heading "Competing interests" to "Disclosure and competing interests statement".

- Please change heading "Acknowledgements and funding" to "Acknowledgements".

- Please change heading "Materials and Methods" to "Methods".

- We use CRediT to specify the contributions of each author in the journal submission system. CRediT replaces the author

contribution section, which should be removed from the manuscript. Please use the free text box to provide more detailed descriptions. See also guide to authors for more information: https://link.springer.com/partners/embo-press/editorial-policies#Authorship.

- We noticed that callouts in the manuscript are missing for "Supplementary Methods Figure 1", which should, however, be renamed to "Appendix Figure S#" (please see also below for more information about the Appendix file and nomenclature).

- Please make sure that the last column of your Author Checklist ("In which section is the information available?") is filled in for every positive response in the "Information included in the manuscript?" column.

- We noticed that supplementary figures S1-S5 have been uploaded both as individual figures and also included in the Appendix file; these figures should either be renamed to Figure EV1-EV5 with their legends included in the manuscript below those of the main Figures, if you wish them to be displayed as Expanded View Figures (please see below for more information), or alternatively they should only be retained in the Appendix file with their legends below each Appendix Figure, using the nomenclature "Appendix Figure S#". In any case, please make sure that all Figure legends and callouts throughout the manuscript are updated as appropriate. Expanded View (EV) files (replacing Supplementary Information) are collapsible/expandable online. A maximum of 5 EV Figures can be typically typeset. EV Figures should be cited as "Figure EV1, Figure EV2" etc. in the text, and their respective legends should be included in the manuscript file after the legends of regular figures. See detailed instructions regarding Expanded View files here: https://link.springer.com/journal/44318/submission-guidelines#expandedview. For the figures that you do NOT wish to display as Expanded View figures, they should be bundled together with their legends in a single PDF file called "Appendix". Appendix figures should be referred to in the main text as: "Appendix Figure S1, Appendix Figure S2" etc. Please see more detailed instructions here: https://link.springer.com/journal/44318/submission-guidelines#expandedview.

- The Appendix file needs to be in PDF format. Its title page should include heading "Appendix for", followed by the manuscript's title (author and affiliation lists are not necessary) and a Table of Contents with page numbers for all listed items. The nomenclature should be "Appendix Figure Sx" and "Appendix Table Sx "throughout the main manuscript file and the Appendix PDF file. Please move the additional references from the Appendix file to the main references list in the manuscript.

- Please note that EMBO press papers are accompanied online by:
A) a short (2 sentences) summary of the findings and their significance,
B) 2-5 short bullet points highlighting the key results, and
C) a synopsis image in .jpg or .png format that is exactly 550 pixels wide and 300 pixels high. Please note that all text needs to be legible at the final size.
Please upload this information along with your revised manuscript (the text for A and B should be provided in a separate Word file).

- Our data editors have checked your Figures and their legends, and raised the following queries. Please address all points below completely in your revised manuscript (all changes should be highlighted or "tracked" in the Word file):
1. Please provide the exact p-values in the legends of Figures 2A, 3D, 4A, S1F.
2. Please note that information related to "n" is missing in the legend of Figure 4A.
3. Please note that the error bars must be defined in the legend of Figure 4A.

- The manuscript sections need to be named and ordered as follows: Title page - Abstract - Introduction - Results - Discussion - Methods - Data Availability - Acknowledgements - Disclosure and Competing Interests Statement - References - Figure Legends - main Tables (if there are any) - Expanded View Figure Legends (if there are any).

- Please also note that as part of the EMBO Press transparent editorial process, The EMBO Journal publishes online a Peer Review File along with each accepted manuscript. This File will be published in conjunction with your paper and will include the referee reports, your point-by-point responses and all pertinent correspondence relating to the manuscript. Your Author's Checklist will also be published at the end of the Peer Review File. Please let us know in case you want to remove any data or figures from your point-by-point responses before they are published as part of the Peer Review File. Retaining unpublished data in the Peer Review File means that these count as published and that the Peer Review File would need to be referenced in future publications. Please let the editorial office know in case you want to remove any data from this file (contact@embojournal.org).

We look forward to seeing a final version of your manuscript as soon as possible. Please let us know if you have any questions and use this link to submit your revision: https://emboj.msubmit.net/cgi-bin/main.plex.

Best regards,

Ioannis

Ioannis Papaioannou, PhD
Editor, The EMBO Journal
i.papaioannou@embojournal.org

Referee #2:

In this revised version , the authors have essentially clarified all points that were raised by this reviewer.
Clearly, the question raised by reviewers 1 and 3 whether this system would indeed by able to generate novel immunogenic targets remains open. Still, I find the concept interesting and surprising to work with relatively low editing levels.

With respect to the answers to reviewer 3, the citation of Uzonyi et al., in combination with the Leaper system is incorrect. Uznyi does show that the positioning of edits depends on mismatches, but the reference for Leaper RNA design is Qu et al, from the Wei lab.

Referee #3:

I believe that this manuscript demonstrates that tumors can be efficiently edited to express candidate targets for therapy.
I believe that all of the points raised by the reviewers have been adequately addressed by the authors.

Response to referees' letter

DEUTSCHES KREBSFORSCHUNGSZENTRUM IN DER HELMHOLTZ-GEMEINSCHAFT

June 19th, 2026

Dear Reviewers,

We thank you for your careful evaluation of our manuscript and for the constructive comments provided throughout the review process. Your suggestions have substantially improved both the quality and clarity of the study.

In this final revision, we have addressed the remaining concern raised by Referee #2 and completed all editorial requirements requested by the journal. We are grateful for your feedback and for the opportunity to improve the manuscript.

With best regards,

Dr. Riccardo Pecori & Prof. Nina Papavasiliou
(for the authors)

Response to Reviewers' comments

Referee #2:

In this revised version , the authors have essentially clarified all points that were raised by this reviewer.

Clearly, the question raised by reviewers 1 and 3 whether this system would indeed by able to generate novel immunogenic targets remains open. Still, I find the concept interesting and surprising to work with relatively low editing levels.

With respect to the answers to reviewer 3, the citation of Uzonyi et al., in combination with the Leaper system is incorrect. Uznyi does show that the positioning of edits depends on mismatches, but the reference for Leaper RNA design is Qu et al, from the Wei lab.

Response: We thank the referee for this correction. The referee is correct that Qu et al. originally described the LEAPER system and its guide RNA design, whereas Uzonyi et al. investigated the influence of mismatch positioning and guide architecture on editing efficiency using LEAPER-derived guide RNAs. Our intention in the response letter was to refer specifically to the effect of the engineered mismatch on editing efficiency rather than to the original LEAPER design. The citation in question appeared only in our previous response letter and not in the manuscript itself. We appreciate the correction and acknowledge the distinction between these two studies.

Referee #3:

I believe that this manuscript demonstrates that tumors can be efficiently edited to express candidate targets for therapy.

I believe that all of the points raised by the reviewers have been adequately addressed by the authors.

Response: We thank the referee for the positive assessment of our revised manuscript and for the constructive feedback provided throughout the review process.

2nd Revision - Editorial Decision 22nd Jun 2026

Dear Nina,

Congratulations on an excellent manuscript! I am very pleased to inform you that it has been accepted for publication in The EMBO Journal. Thank you for comprehensively addressing the initially raised referees' concerns and our editorial requests for changes and corrections.

Your manuscript will be processed for publication by EMBO Press. It will be copy edited and you will receive page proofs prior to publication. Please note that you will be contacted by Springer Nature Author Services to complete licensing and payment information.

You may qualify for financial assistance for your publication charges - either via a Springer Nature fully open access agreement or an EMBO initiative. Check your eligibility: https://link.springer.com/journal/44318/how-to-publish-with-us

Should you be planning a Press Release on your article, please notify the production team via this form in order to coordinate publication and release dates: https://forms.cloud.microsoft/e/eH4A8JVP6g

If you have any questions, please do not hesitate to contact the Editorial Office. Thank you for your contribution to The EMBO Journal. Working with you has been a pleasure!

Best regards,

Ioannis

Ioannis Papaioannou, PhD
Editor, The EMBO Journal
i.papaioannou@embojournal.org

*****
Please note that it is The EMBO Journal policy for the transcript of the editorial process (containing referee reports and your response letters) to be published as an online supplement to each paper. If you should prefer removal of any referee-only figures included in the point-by-point response(s), e.g. because they may still be used for future publication or because they have been reproduced from published work by others, please do let us know immediately via response email.
More information is available here: https://link.springer.com/partners/embo-press/editorial-policies#Peer%20review

** Click here to be directed to your login page: https://emboj.msubmit.net

EMBO Press Author Checklist

Corresponding Author Name: F. Nina Papavasiliou	
Journal Submitted to: The EMBO Journal	
Manuscript Number: EMBOJ-2025-123135R	

USEFUL LINKS FOR COMPLETING THIS FORM
The EMBO Journal - Author Guidelines
EMBO Reports - Author Guidelines
Molecular Systems Biology - Author Guidelines
EMBO Molecular Medicine - Author Guidelines

Reporting Checklist for Life Science Articles (updated January
This checklist is adapted from Materials Design Analysis Reporting (MDAR) Checklist for Authors. MDAR establishes a minimum set of requirements in transparent reporting in the life sciences (see Statement of Task: 10.31222/osf.io/9sm4x). Please follow the journal's guidelines in preparing your
Please note that a copy of this checklist will be published alongside your article.

Abridged guidelines for figures
1. Data
The data shown in figures should satisfy the following conditions:
- the data were obtained and processed according to the field's best practice and are presented to reflect the results of the experiments in an accurate and unbiased manner.
- ideally, figure panels should include only measurements that are directly comparable to each other and obtained with the same assay.
- plots include clearly labeled error bars for independent experiments and sample sizes. Unless justified, error bars should not be shown for technical
- if n<5, the individual data points from each experiment should be plotted. Any statistical test employed should be justified.
- Source Data should be included to report the data underlying figures according to the guidelines set out in the authorship guidelines on Data

2. Captions
Each figure caption should contain the following information, for each panel where they are relevant:
- a specification of the experimental system investigated (eg cell line, species name).
- the assay(s) and method(s) used to carry out the reported observations and measurements.
- an explicit mention of the biological and chemical entity(ies) that are being measured.
- an explicit mention of the biological and chemical entity(ies) that are altered/varied/perturbed in a controlled manner.
- the exact sample size (n) for each experimental group/condition, given as a number, not a range;
- a description of the sample collection allowing the reader to understand whether the samples represent technical or biological replicates (including how many animals, litters, cultures, etc.).
- a statement of how many times the experiment shown was independently replicated in the laboratory.
- definitions of statistical methods and measures:
  - common tests, such as t-test (please specify whether paired vs. unpaired), simple χ2 tests, Wilcoxon and Mann-Whitney tests, can be unambiguously identified by name only, but more complex techniques should be described in the methods section;
  - are tests one-sided or two-sided?
  - are there adjustments for multiple comparisons?
  - exact statistical test results, e.g., P values = x but not P values < x;
  - definition of 'center values' as median or average;
  - definition of error bars as s.d. or s.e.m.

Please complete ALL of the questions below.
Select "Not Applicable" only when the requested information is not relevant for your study.

Materials

Newly Created Materials	Information included in the manuscript?	In which section is the information available? (Reagents and Tools Table, Materials and Methods, Figures, Data Availability Section)
New materials and reagents need to be available; do any restrictions apply?	Yes	Methods

Antibodies	Information included in the manuscript?	In which section is the information available? (Reagents and Tools Table, Materials and Methods, Figures, Data Availability Section)
For **antibodies** provide the following information:<br>- Commercial antibodies: RRID (if possible) or supplier name, catalogue number and or/clone number<br>- Non-commercial: RRID or citation	Yes	Methods

DNA and RNA sequences	Information included in the manuscript?	In which section is the information available? (Reagents and Tools Table, Materials and Methods, Figures, Data Availability Section)
**Short novel DNA or RNA including primers, probes**: provide the sequences.	Yes	Methods, supplemetary information

Cell materials	Information included in the manuscript?	In which section is the information available? (Reagents and Tools Table, Materials and Methods, Figures, Data Availability Section)
**Cell lines:** Provide species information, strain. Provide accession number in repository **OR** supplier name, catalog number, clone number, and/**OR** RRID.	Yes	Methods
**Primary cultures:** Provide species, strain, sex of origin, genetic modification status.	Not Applicable	
Report if the cell lines were recently **authenticated** (e.g., by STR profiling) and tested for mycoplasma contamination.	Yes	Material and Methods, while the cell lines were authenticated recently we do not provide the reports.

Experimental animals	Information included in the manuscript?	In which section is the information available? (Reagents and Tools Table, Materials and Methods, Figures, Data Availability Section)
**Laboratory animals or Model organisms:** Provide species, strain, sex, age, genetic modification status. Provide accession number in repository **OR** supplier name, catalog number, clone number, **OR** RRID.	Yes	Methods
**Animal observed in or captured from the field:** Provide species, sex, and age where possible.	Not Applicable	
Please detail **housing and husbandry conditions**.	Yes	Methods

Plants and microbes	Information included in the manuscript?	In which section is the information available? (Reagents and Tools Table, Materials and Methods, Figures, Data Availability Section)
**Plants:** provide species and strain, ecotype and cultivar where relevant, unique accession number if available, and source (including location for collected wild specimens).	Not Applicable	
**Microbes:** provide species and strain, unique accession number if available, and source.	Not Applicable	

Human research participants	Information included in the manuscript?	In which section is the information available? (Reagents and Tools Table, Materials and Methods, Figures, Data Availability Section)
If collected and within the bounds of privacy constraints report on age, sex and gender or ethnicity for all study participants.	Not Applicable	

Core facilities	Information included in the manuscript?	In which section is the information available? (Reagents and Tools Table, Materials and Methods, Figures, Data Availability Section)
If your work benefited from core facilities, was their service mentioned in the acknowledgments section?	Yes	Acknowledgments

Design

Study protocol	Information included in the manuscript?	In which section is the information available? (Reagents and Tools Table, Materials and Methods, Figures, Data Availability Section)
If study protocol has been **pre-registered, provide DOI in the manuscript**. For clinical trials, provide the trial registration number **OR** cite DOI.	Not Applicable	
Report the **clinical trial registration number** (at ClinicalTrials.gov or equivalent), where applicable.	Not Applicable	

Laboratory protocol	Information included in the manuscript?	In which section is the information available? (Reagents and Tools Table, Materials and Methods, Figures, Data Availability Section)
Provide DOI OR other citation details if **external detailed step-by-step protocols** are available.	Yes	Methods

Experimental study design and statistics	Information included in the manuscript?	In which section is the information available? (Reagents and Tools Table, Materials and Methods, Figures, Data Availability Section)
Include a statement about **sample size** estimate even if no statistical methods were used.	Not Applicable	
Were any steps taken to minimize the effects of subjective bias when allocating animals/samples to treatment (e.g. **randomization procedure**)? If yes, have they been described?	Not Applicable	
Include a statement about **blinding** even if no blinding was done.	Not Applicable	
Describe **inclusion/exclusion criteria** if samples or animals were excluded from the analysis. Were the criteria pre-established?<br><br>If sample or data points were omitted from analysis, report if this was due to attrition or intentional exclusion and provide justification.	Not Applicable	
For every figure, are **statistical tests** justified as appropriate? Do the data meet the assumptions of the tests (e.g., normal distribution)? Describe any methods used to assess it. Is there an estimate of variation within each group of data? Is the variance similar between the groups that are being statistically compared?	Yes	Methods, Figure legends, Supplementary Information

Sample definition and in-laboratory replication	Information included in the manuscript?	In which section is the information available? (Reagents and Tools Table, Materials and Methods, Figures, Data Availability Section)
In the figure legends: state number of times the experiment was **replicated** in laboratory.	Yes	Figure legends
In the figure legends: define whether data describe **technical or biological replicates**.	Yes	Figure legends

Ethics

Ethics	Information included in the manuscript?	In which section is the information available? (Reagents and Tools Table, Materials and Methods, Figures, Data Availability Section)
Studies involving **human participants**: State details of **authority granting ethics approval** (IRB or equivalent committee(s), provide reference number for approval.	Not Applicable	
Studies involving **human participants**: Include a statement confirming that **informed consent** was obtained from all subjects and that the experiments conformed to the principles set out in the WMA Declaration of Helsinki and the Department of Health and Human Services Belmont Report.	Not Applicable	
Studies involving **human participants:** For publication of **patient photos**, include a statement confirming that consent to publish was obtained.	Not Applicable	
Studies involving experimental **animals**: State details of **authority granting ethics approval** (IRB or equivalent committee(s), provide reference number for approval. Include a statement of compliance with ethical regulations.	Yes	Methods
Studies involving **specimen and field samples:** State if relevant **permits** obtained, provide details of authority approving study; if none were required, explain why.	Not Applicable	

Dual Use Research of Concern (DURC)	Information included in the manuscript?	In which section is the information available? (Reagents and Tools Table, Materials and Methods, Figures, Data Availability Section)
Could your study fall under dual use research restrictions? Please check biosecurity documents and list of **select agents and toxins** (CDC): https://www.selectagents.gov/sat/list.htm	Not Applicable	
If you used a select agent, is the security level of the lab appropriate and reported in the manuscript?	Not Applicable	
If a study is subject to dual use research of concern regulations, is the name of the **authority granting approval and reference number** for the regulatory approval provided in the manuscript?	Not Applicable	

Reporting

The MDAR framework recommends adoption of discipline-specific guidelines, established and endorsed through community initiatives. Journals have their own policy about requiring specific guidelines and recommendations to complement MDAR.

Adherence to community standards	Information included in the manuscript?	In which section is the information available? (Reagents and Tools Table, Materials and Methods, Figures, Data Availability Section)
State if relevant guidelines or checklists (e.g., **ICMJE, MIBBI, ARRIVE, PRISMA**) have been followed or provided.	Not Applicable	
For **tumor marker prognostic studies,** we recommend that you follow the **REMARK** reporting guidelines (see link list at top right). See author guidelines, under 'Reporting Guidelines'. Please confirm you have followed these guidelines.	Not Applicable	
For **phase II and III randomized controlled trials**, please refer to the **CONSORT** flow diagram (see link list at top right) and submit the CONSORT checklist (see link list at top right) with your submission. See author guidelines, under 'Reporting Guidelines'. Please confirm you have submitted this list.	Not Applicable	

Data Availability

Data availability	Information included in the manuscript?	In which section is the information available? (Reagents and Tools Table, Materials and Methods, Figures, Data Availability Section)
Have **primary datasets** been deposited according to the journal's guidelines (see 'Data Deposition' section) and the respective accession numbers provided in the Data Availability Section?	Yes	Data Availability Section
Were **human clinical and genomic datasets** deposited in a public access-controlled repository in accordance to ethical obligations to the patients and to the applicable consent agreement?	Not Applicable	
Are **computational models** that are central and integral to a study available without restrictions in a machine-readable form? Were the relevant accession numbers or links provided?	Yes	Data Availability Section
If publicly available data were reused, provide the respective **data citations in the reference list**.	Yes	Manuscript and Reference list
