# Supplementary material for: Engineering editopes through programmable RNA editing toward tumor neoantigen generation
Source: EMBO J. 2026 Aug 14;45(18):6655–78. doi: 10.1038/s44318-026-00870-5 (PMC13578223; doi:10.1038/s44318-026-00870-5)
Supplement: Supplementary file 3 [file 44318_2026_870_MOESM8_ESM.pdf]

Expanded View Figures

Figure EV1. Optimization of Short Precise-Encodable ADAR Recruiting (SPEAR) gRNAs.

(**A**) Schematic of the $eGFP^{W58X}$ target mRNA and the corresponding targeting gRNAs used in the experiment shown in panel (**B**). (**B**) Flow cytometry analysis of mCherry and eGFP double-positive cells, presented as a bar plot for the tested gRNAs. Bars represent the mean for three biological replicates; error bars indicate the standard deviation. (**C**) Diagram illustrating the difference between LEAPER gRNAs without (left (Qu et al, 2019)) or with a 4-nucleotide (nt) mismatch (right (Uzonyi et al, 2021)). (**D**) Schematic of the sequencing window where A-to-I edits were assessed, as shown in panel (**E**). (**E**) Heatmap of A-to-I editing from NGS amplicon sequencing, showing on-target editing (in blue) and off-target editing (in red) for three different LEAPER gRNAs. The region targeted by each gRNA is indicated with a red rectangle. (**F**) Flow cytometry analysis of mCherry- and eGFP double-positive cells (left, gray bars) and eBFP-positive cells (right, blue bars), presented as a bar plot for the optimized SPEAR gRNA in HEK293T ADAR1 WT and KO dual-reporter cell lines. Bars represent the mean for three biological replicates; error bars indicate the standard deviation (adjusted *p* values: **** <0.0001, ns = 0.3928, two-way ANOVA with multiple comparisons). (**G**) Flow cytometry analysis of mCherry expression of HEK293T ADAR1 WT and KO dual-reporter cell lines, used in the experiment shown in panel (**F**).

© The Author(s)

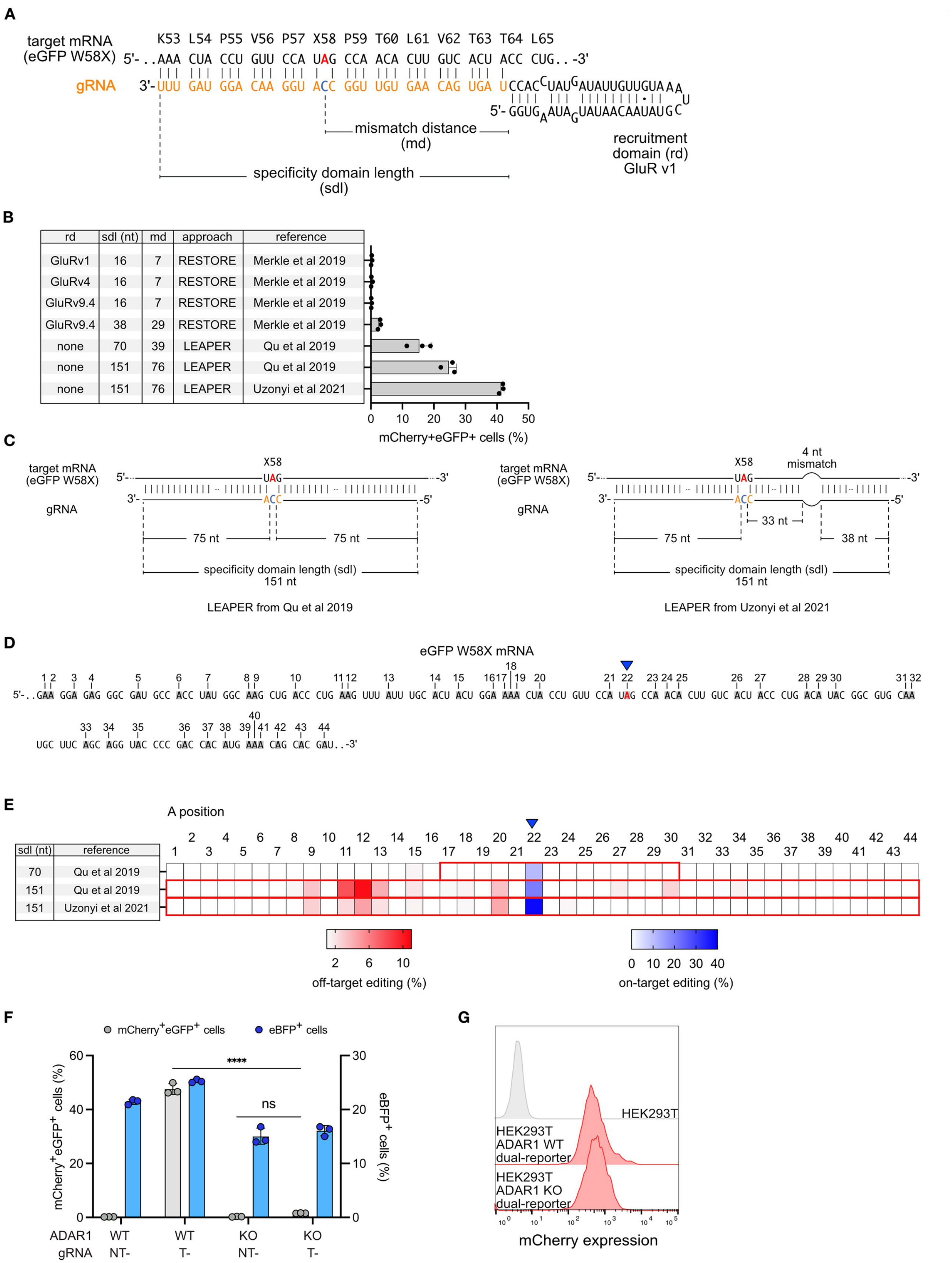
A
target mRNA (eGFP W58X)
K53 L54 P55 V56 P57 X58 P59 T60 L61 V62 T63 T64 L65
5'-..AAA CUA CCU GUU CCA UAG CCA ACA CUU GUC ACU ACC CUG..-3'
gRNA
3'-UUU GAU GGA CAA GGU ACC GGU UGU GAA CAG UGA U
mismatch distance (md)
specificity domain length (sdl)
recruitment domain (rd) GluR v1
B
rd | sdl (nt) | md | approach | reference
GluRv1 | 16 | 7 | RESTORE | Merkle et al 2019
GluRv4 | 16 | 7 | RESTORE | Merkle et al 2019
GluRv9.4 | 16 | 7 | RESTORE | Merkle et al 2019
GluRv9.4 | 38 | 29 | RESTORE | Merkle et al 2019
none | 70 | 39 | LEAPER | Qu et al 2019
none | 151 | 76 | LEAPER | Qu et al 2019
none | 151 | 76 | LEAPER | Uzonyi et al 2021
mCherry+eGFP+ cells (%)
C
target mRNA (eGFP W58X)
gRNA
X58
75 nt
75 nt
specificity domain length (sdl) 151 nt
LEAPER from Qu et al 2019
4 nt mismatch
33 nt
38 nt
LEAPER from Uzonyi et al 2021
D
eGFP W58X mRNA
E
A position
sdl (nt) | reference
70 | Qu et al 2019
151 | Qu et al 2019
151 | Uzonyi et al 2021
off-target editing (%)
on-target editing (%)
F
mCherry+eGFP+ cells
eBFP+ cells
mCherry+eGFP+ cells (%)
eBFP+ cells (%)
ns
ADAR1 WT WT KO KO
gRNA NT- T- NT- T-
G
HEK293T
HEK293T ADAR1 WT dual-reporter
HEK293T ADAR1 KO dual-reporter
mCherry expression

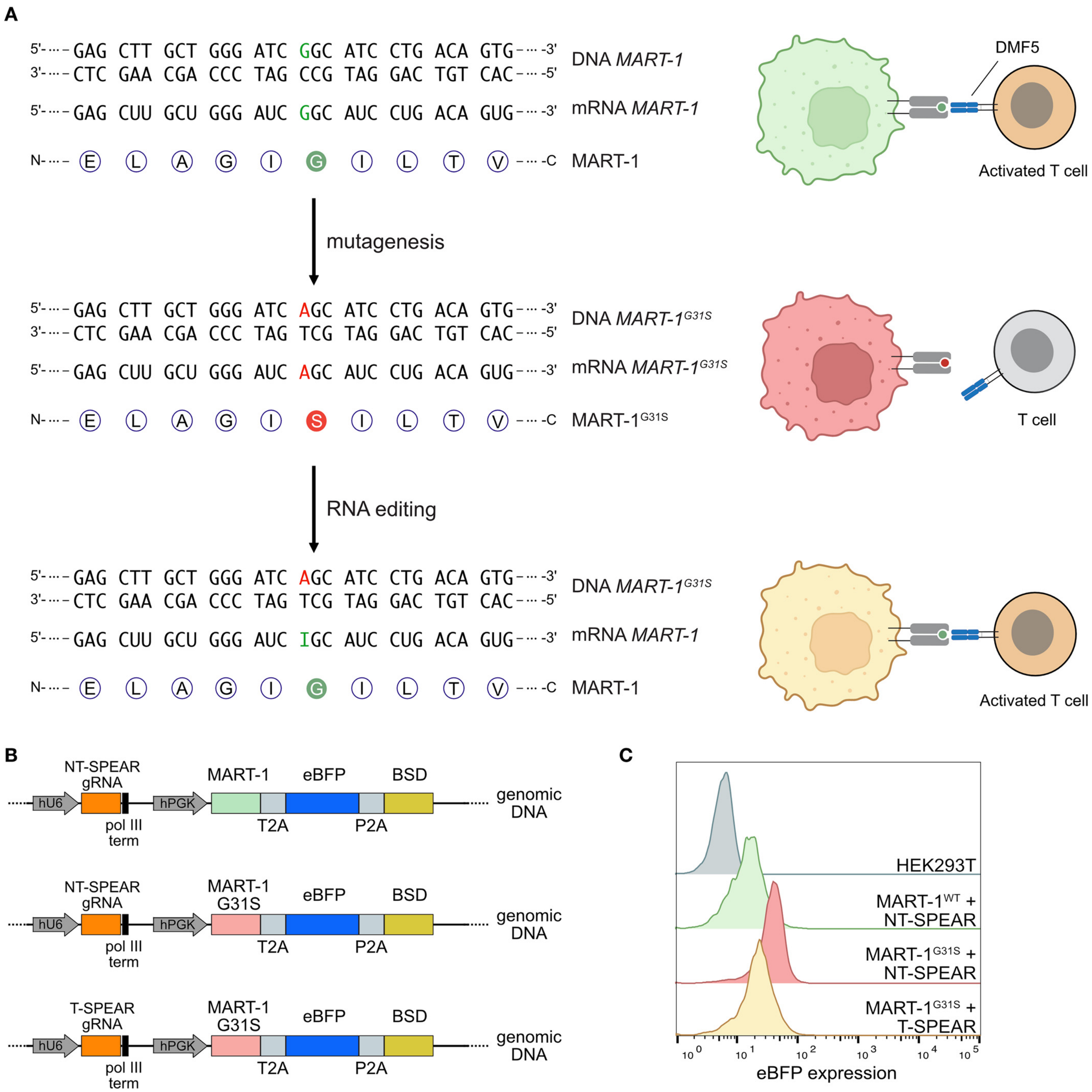

Figure EV2. In vitro T cell activation using SPEAR gRNAs.

(**A**) Diagram illustrating the molecular setup (left) and the predicted outcome of the cellular coculture (right) corresponding to the experiment presented in Fig. 2. (**B**) Schematic of the constructs used to generate HEK293T cells expressing either wild-type (WT) or G31S mutant MART-1, along with either a non-targeting (NT) or targeting (T) SPEAR gRNA. eBFP was used as a reporter for MART-1 (WT or G31S) expression, and a blasticidin resistance gene (BSD) was used for selecting transfected cells. (**C**) Flow cytometry analysis of eBFP expression for the different cell lines.

 © The Author(s)

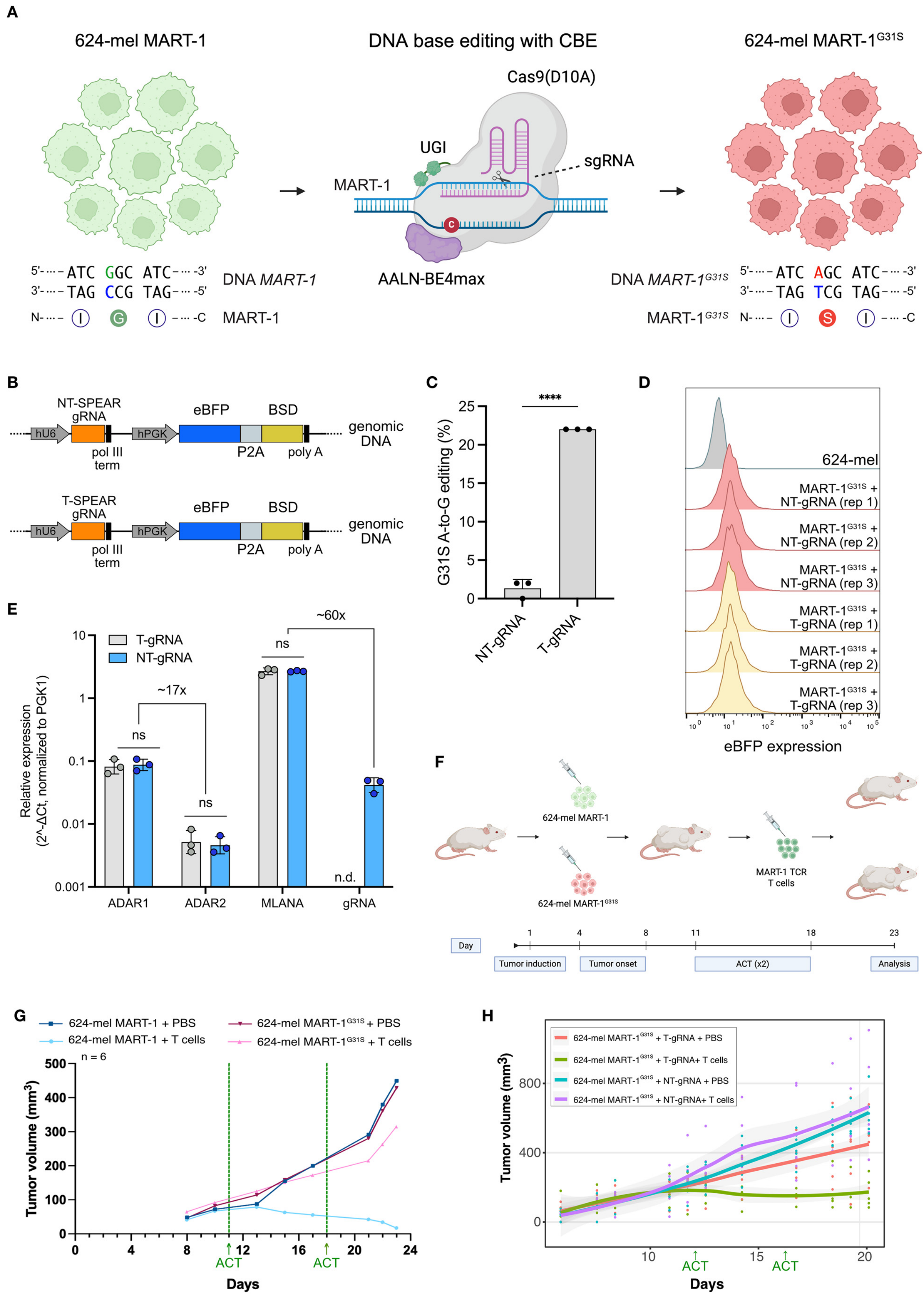
A
624-mel MART-1
DNA base editing with CBE
624-mel MART-1G31S
Cas9(D10A)
UGI
sgRNA
MART-1
AALN-BE4max
5'-···- ATC GGC ATC -···-3'
3'-···- TAG CCG TAG -···-5'
DNA MART-1
N-··· - I G I - ··· -C MART-1
DNA MART-1G31S
5'-···- ATC AGC ATC -···-3'
3'-···- TAG TCG TAG -···-5'
MART-1G31S N-··· - I S I - ··· -C
B
NT-SPEAR gRNA
T-SPEAR gRNA
hU6
hPGK
eBFP
BSD
pol III term
P2A
poly A
genomic DNA
C
G31S A-to-G editing (%)
****
NT-gRNA
T-gRNA
D
624-mel
MART-1G31S + NT-gRNA (rep 1)
MART-1G31S + NT-gRNA (rep 2)
MART-1G31S + NT-gRNA (rep 3)
MART-1G31S + T-gRNA (rep 1)
MART-1G31S + T-gRNA (rep 2)
MART-1G31S + T-gRNA (rep 3)
eBFP expression
E
T-gRNA
NT-gRNA
Relative expression (2^-ΔCt, normalized to PGK1)
~17x
~60x
ns
n.d.
ADAR1
ADAR2
MLANA
gRNA
F
624-mel MART-1
624-mel MART-1G31S
MART-1 TCR T cells
Day
Tumor induction
Tumor onset
ACT (x2)
Analysis
G
624-mel MART-1 + PBS
624-mel MART-1 + T cells
624-mel MART-1G31S + PBS
624-mel MART-1G31S + T cells
n = 6
Tumor volume (mm3)
ACT
Days
H
624-mel MART-1G31S + T-gRNA + PBS
624-mel MART-1G31S + T-gRNA+ T cells
624-mel MART-1G31S + NT-gRNA + PBS
624-mel MART-1G31S + NT-gRNA+ T cells
Tumor volume (mm3)
ACT
Days

© The Author(s)

Figure EV3. Generation of 624-mel mutant and edited cell lines for evaluating T cell activation and tumor cell elimination in vivo.

(**A**) Schematic of the procedure used to generate 624-mel cell clones endogenously expressing MART-1^{G31S}. The cytosine base editor AALN-BE4max(Doman et al, 2020) was used to target the cytosine (C highlighted in blue) on the template strand of DNA, opposite the first guanine (G highlighted in green) in the GGC codon encoding glycine (G) at position 31 of the *MART-1* gene. C-to-U base editing converted the codon to AGC, resulting in a glycine-to-serine substitution (G31S). (**B**) Schematic of the constructs used to generate 624-mel MART-1^{G31S} cells expressing either a non-targeting (NT) or targeting (T) SPEAR gRNA. eBFP was used as a reporter for plasmid integration, and a blasticidin resistance gene (BSD) was used for selection. (**C**) Bar plot showing average A-to-I editing percentages at the MART-1 G31S site, quantified from Sanger sequencing traces using MultiEditR (Kluesner et al, 2021) in 624-mel MART-1^{G31S} cells transiently transfected with the plasmids shown in **B** (*p* value: **** <0.0001, two-tailed unpaired *t*-test). Bars represent the mean for three biological replicates; error bars indicate the standard deviation. (**D**) Flow cytometry analysis of eBFP expression in 624-mel MART-1^{G31S} expressing either NT- or T-SPEAR gRNAs, used in the experiments shown in Fig. 4. (**E**) Bar plot representing the expression of ADAR1, ADAR2, MLANA and gRNA measured by RT-qPCR in the 624-mel MART-1^{G31S} lines expressing either NT- or T-SPEAR gRNAs. Relative expression is normalized to PGK1. A two-tailed unpaired *t*-test with Welch's correction was used to compare the differences in delta Ct values. ns = not significant, center = mean and error bars = standard deviation, $N = 3$. (**F**) Schematic of the in vivo experimental setup for the results shown in (**G**). (**G**) Tumor growth analysis of 624-mel cells expressing MART-1 or MART-1^{G31S} with or without adoptive cell therapy (ACT) using MART-1-specific human-derived T cells. (**H**) Alternative visualization of the in vivo tumor growth shown in Fig. 4E. Each curve represents the mean of six mice, and every single dot represents each mouse.

© The Author(s)
