# Supplementary material for: Engineering editopes through programmable RNA editing toward tumor neoantigen generation
Source: EMBO J. 2026 Aug 14;45(18):6655–78. doi: 10.1038/s44318-026-00870-5 (PMC13578223; doi:10.1038/s44318-026-00870-5)
Supplement: Supplementary file 5 [file 44318_2026_870_MOESM4_ESM.zip › Figure2_data/README_Figure2.rtf]

The file “Figure2A_data” contains the editing values used for the bar plot shown in 2A. Editing was detected from Sanger traces (provided within the folder “Figure2B_data”) using MultiEditR. The same Sanger traces were used for panel B.
