# Supplementary material for: Engineering editopes through programmable RNA editing toward tumor neoantigen generation
Source: EMBO J. 2026 Aug 14;45(18):6655–78. doi: 10.1038/s44318-026-00870-5 (PMC13578223; doi:10.1038/s44318-026-00870-5)
Supplement: Supplementary file 6 [file 44318_2026_870_MOESM5_ESM.zip › Figure3_data/README_Figure3.rtf]

The folder “Figure3B_data” contains the representative Sanger traces used in panel B.

The folder “Figure3C_data” contains the raw FCS file for the flow cytometry analysis and the FlowJo analysis file. 

The names of the files used are based on the following plasmid numbers:
- 468 = NT-gRNA, coMART1-WT
- 469 = NT-gRNA, coMART1-G31S
- 500 = T-gRNA, coMART1-G31S

The folder “Figure3D_data” contains two Excel sheets with all the numbers used for panel D. Additionally, the file “Figure 3D_Raw data Immunopeptidomic analysis” contains all the data needed for the quantification.
