# Supplementary material for: Engineering editopes through programmable RNA editing toward tumor neoantigen generation
Source: EMBO J. 2026 Aug 14;45(18):6655–78. doi: 10.1038/s44318-026-00870-5 (PMC13578223; doi:10.1038/s44318-026-00870-5)
Supplement: Supplementary file 7 [file 44318_2026_870_MOESM6_ESM.zip › Figure4_data/Figure4A_data/README_Figure4A.rtf]

The file “Figure4A_MultiEditR editing analysis” contains the editing values used for the bar plot shown in 4A. Editing was detected from Sanger traces (provided within the folder “Figure4A_Sanger Traces_RT-PCRs”) using MultiEditR. This Excel sheet also contains the parameters used in MultiEditR for editing detection.
