# Supplementary material for: Engineering editopes through programmable RNA editing toward tumor neoantigen generation
Source: EMBO J. 2026 Aug 14;45(18):6655–78. doi: 10.1038/s44318-026-00870-5 (PMC13578223; doi:10.1038/s44318-026-00870-5)
Supplement: Supplementary file 7 [file 44318_2026_870_MOESM6_ESM.zip › Figure4_data/Figure4B_data/README_Figure4B.rtf]

This folder contains two additional folders:
1 - “Figure4B_Sanger Traces_cDNA”
2 - “Figure4B_Sanger Traces_DNA”

Folder 1 contains the Sanger traces used in the two lower represetative sanger tarces for cDNA used in Figure 4B.

Folder 2 contains Sanger traces from all samples represented in Figure A and B upon PCR amplification from genomic DNA. The one trace represented in the top of Figure 4B (DNA T-gRNA) is “2025-02-20_AA25_08 Q5 RP640 Rep001_BC1486_T-gRNA”.
